# Protein Digests and Pure Peptides from Chia Seed Prevented Adipogenesis and Inflammation by Inhibiting PPARγ and NF-κB Pathways in 3T3L-1 Adipocytes

**DOI:** 10.3390/nu13010176

**Published:** 2021-01-08

**Authors:** Mariana Grancieri, Hércia Stampini Duarte Martino, Elvira Gonzalez de Mejia

**Affiliations:** 1 Departamento de Nutrição e Saúde, Universidade Federal de Viçosa, Viçosa, MG 36570-900, Brazil; marianagrancieri@gmail.com (M.G.); hercia72@gmail.com (H.S.D.M.); 2 Department of Food Science & Human Nutrition, University of Illinois at Urbana-Champaign, Urbana, IL 61801, USA

**Keywords:** adipocytes, bioactive peptides, digested proteins, inflammation, obesity, *Salvia hispanica* L.

## Abstract

The objective was to evaluate the mechanisms of digested total proteins (DTP), albumin, glutelin, and pure peptides from chia seed (*Salvia hispanica* L.) to prevent adipogenesis and its associated inflammation in 3T3-L1 adipocytes. Preadipocytes (3T3-L1) were treated during differentiation with either DTP or digested albumin or glutelin (1 mg/mL) or pure peptides NSPGPHDVALDQ and RMVLPEYELLYE (100 µM). Differentiated adipocytes also received DTP, digested albumin or glutelin (1 mg/mL), before (prevention) or after (inhibition) induced inflammation by addition of conditioned medium (CM) from inflamed macrophages. All treatments prevented adipogenesis, reducing more than 50% the expression of PPARγ and to a lesser extent lipoprotein lipase (LPL), fatty acid synthase (FAS), sterol regulatory element-binding protein 1 (SREBP1), lipase activity and triglycerides. Inflammation induced by CM was reduced mainly during prevention, while DTP decreased expression of NF-κB (−48.4%), inducible nitric oxide synthase (iNOS) (−46.2%) and COX-2 (−64.5%), *p* < 0.05. Secretions of nitric oxide, PGE2 and TNFα were reduced by all treatments, *p* < 0.05. DTP reduced expressions of iNOS (−52.1%) and COX-2 (−66.4%). Furthermore, digested samples and pure peptides prevented adipogenesis by modulating PPARγ and additionally, preventing and even inhibiting inflammation in adipocytes by inhibition of PPARγ and NF-κB expression. These results highlight the effectiveness of digested total proteins and peptides from chia seed against adipogenesis complications in vitro.

## 1. Introduction

A major cause of death and premature incapacity worldwide is cardiovascular diseases (CVD), representing around 17.3 million deaths per year [1]. An important modifiable risk factor for coronary heart disease is obesity [2]. It is estimated that over half of the global adult population is overweight or obese [3]. Obesity is characterized by expansion of adipose tissue starting by adipocyte hyperplasia, mediated by the recruitment and proliferation of adipogenic precursors, mainly the peroxisome proliferator-activated receptor gamma (PPARγ), followed by adipocyte hypertrophy [4].

Adipose tissue is not only a fat deposit in the body. It is also an endocrine organ that synthesizes and releases into systemic circulation adipokines, such as leptin, resistin and adiponectin hormones, chemokines and cytokines [5]. This condition induces infiltration of activated macrophages into the adipose tissue that releases pro-inflammatory cytokines, as tumor necrosis factor alpha (TNF-α), interleukin-6 (IL-6) and interleukin-1β (IL-1β), which increase local inflammation and stimulate pro-inflammatory secretion creating a vicious cycle of the inflammatory response [6,7]. This scenario, known as low-grade inflammation is associated with an overall impact on obesity-related complications, like insulin resistance, progression to type 2 diabetes, cardiovascular disease, metabolic syndrome, and cancer [8].

Obesity is caused by changes in lifestyle, including overconsumption of food and decreased physical activity [9]. Therefore, treatment to reduce obesity consists of lifestyle changes and reduction of energy intake [10]. The consumption of proteins from vegetables has been inversely related to development and progression of obesity by affecting satiety, thermogenesis, energy efficiency and body composition [11]. A vegetable protein source is chia seed (*Salvia hispanica* L.). Chia is an herbaceous plant native to northern Guatemala and southern Mexico, which produces small seeds that stand out due to their high nutritional and functional value [12]. They are also related with beneficial health effects as indicated by lower markers of obesity, diabetes, cancer, dyslipidemia, and CVD [13,14,15,16,17,18].

Chia seed has about 20% protein [19]. Main storage protein fractions in chia are prolamin, glutelin, albumin, and globulin, being the last two in higher concentrations [20,21]. After gastrointestinal digestion they generate peptides that, according to their composition and amino acid sequence, may exert antimicrobial, antihypertensive, hypocholesterolemic, antithrombotic, antioxidant, increased absorption/bioavailability of minerals and immunomodulatory effects, among others [22,23].

Research has shown that digested protein of chia inhibits ACE-enzyme (angiotensin-converting enzyme) activity [21,23], has high antioxidant and antibacterial effects [13,24,25,26], shows inhibition of cholesterol synthesis [24]. A recent research from our group showed that digested total protein (DTP), digested albumin, globulin and glutelin from chia seed prevented against inflammation and atherosclerosis process in macrophages RAW 264.7 [25]. However, there is no evidence about the effect of digested proteins and pure peptides from chia seed on adipogenesis and inflammation induction to adipose tissue and the mechanisms of action. The objectives of this research were to evaluate and compare the in vitro mechanisms of digested total proteins, albumin, glutelin and pure peptides from chia seed (*Salvia hispanica* L.) to prevent adipogenesis and its associated inflammation in 3T3-L1 adipocytes. The hypothesis was that digested total proteins, protein fractions and pure peptides from chia seed will block PPARγ expression and several markers related to the reduction in the development of adipogenesis in 3T3-L1 adipocytes. Additionally, the digested chia proteins prevented and inhibited inflammation, induced by conditioned medium from inflamed macrophages, by blocking PPARγ and, consequently, nuclear factor-kappa B (NF-κB) expression.

## 2. Materials and Methods

### 2.1. Materials

3T3-L1 and RAW 264.7 cells were purchased from American Type Culture Collection (ATCC, Manassas, VA, USA). The Dulbecco’s modified Eagle medium (DMEM) was purchased from Corning Cellgro (Manassas, VA, USA), fetal bovine serum (FBS), newborn calf serum (NCS) and penicillin-streptomycin (100×) were obtained from Gibco Life Technologies (Grand Island, NY, USA). Primary antibodies NF-κB p65 (sc-8008), FAS (sc-48357), LPL (sc-373759), PPARγ (sc-7273), SREBP-1 (sc-365513) and RIPA Lysis Buffer System were obtained from Santa Cruz Biotechnology (Santa Cruz, CA, USA). Primary antibodies COX-2 (MA5-14568), glyceraldehyde 3-phosphate dehydrogenase (GAPDH) (MA5-15738), 2′,7′-dichlorodihydrofluorescein diacetate (H_2_DCFDA) (D339) and the pure peptides were obtained from Thermo Fisher Scientific (Rockford, IL, USA) with >98% of purity. All other chemicals and reagents were obtained from Sigma-Aldrich (St. Louis, MO, USA) unless otherwise specified.

### 2.2. Preparation of Digested Total Protein and Protein Fractions

The chia seeds obtained from Rio Grande do Sul/Brazil were prepared as described by Orona-Tamayo et al. [21] with modifications. Total protein was extracted as detailed in Grancieri, Martino, Gonzalez de Mejia [19]. Briefly, deionized water was added to the mucilage and fat-free chia flour (1:20, g:mL), the pH was adjusted to 8.0 and placed under constant stirring (35 °C, 1 h). After, the mixture was centrifuged (5000× *g*; 15 min; 25 °C) and the supernatant collected, freeze-dried and stored at −20 °C.

The storage protein fractions albumin and glutelin were obtained by the solubility method according to Osbore [26] classification by Orona-Tamayo et al. [21]. In this, mucilage-free and fat-free chia flour were diluted with deionized water (1:10, g:mL), mixed for 1 h at 4 °C, centrifuged (14,000× *g*; 20 min; 4 °C) and the supernatant was labeled as the albumin fraction. The resulting precipitate was resuspended with 0.05 mol L^−1^ Tris-hydrochloric acid (HCl) + 0.5 mol/l sodium chloride (NaCl) (pH 8) (1:10, g:mL), mixed, centrifuged, as above and the supernatant defined as globulin fraction. The pellet was diluted with isopropanol 70% (1:10, g:mL), processed as above and the supernatant was labeled as prolamin fraction. Then, the resulting pellet was added with 0.1 mol/L sodium tetraborate decahydrate (Na_2_B_4_O_7_-H_2_O) (pH 10) (1:10, g:mL), processed as above and the supernatant was named as glutelin fraction. All samples were freeze-dried, stored at −20 °C and used within 7 months. Based on the sodium dodecyl sulphate–polyacrylamide gel electrophoresis (SDS-PAGE) pattern of the bands obtained (data not shown), these were similar to those of other publication that also extracted these fractions from chia [21].

All proteins were freeze-dried and submitted to simulated gastrointestinal digestion using the adapted procedure outlined by Megias et al. [27]. Briefly, each sample was suspended in deionized water (1:20 g:mL) and digested with pepsin (pH 2.0) followed by pancreatin (pH 7.5), during 1h at 37 °C, each one. The process was stopped by heating in water bath (75 °C, 20 min) and the proteins were centrifuged twice at 20,000× *g*, 15 min, 4 °C. The supernatant was dialyzed using a 100–500 Da molecular weight cut-off membrane (Spectra/Por, Biotech Cellulose Ester Membrane), freeze-dried and stored at −20 °C until analysis (Figure S1).

### 2.3. Identification, Characterization of Potentially Bioactive Peptides

The resulting peptides from DTP (digested total protein) and digested albumin and glutelin were analyzed by high-performance liquid chromatography–electrospray ionization–mass spectrometry (HPLC–ESI–MS) using a Q-ToF Ultima mass spectrometer (Waters, Milford, MA, USA), according to Mojica, Chen, de Mejia [28]. The sequence of amino acids was identified using MassLynx version V4.1 software (Waters Corp., Milford, MA, USA); no images were created in this study using the MassLynx V4.1 software. The peptides with more than 90% of probability had their biological activity predicted by BIOPEP database (http://www.uwm.edu.pl/biochemia/index.php/pl/biopep, accessed on 27 February 2018).

Peptides NSPGPHDVALDQ and RMVLPEYELLYE from glutelin fraction were selected by their high relative abundance on chromatogram (34.6% and 92.2%, respectively), antioxidant activity (predicted by BIOPEP database), low hydrophobicity and the presence of antioxidant and certain hydrophobic amino acids. The peptide NSPGPHDVALDQ was called as Pep1 and RMVLPEYELLYE as Pep2. The pure peptides were purchase from Thermo Fisher Scientific (Rockford, IL, USA) with >98% of purity.

### 2.4. Cell Culture

3T3-L1 preadipocytes were grown in DMEM supplemented with 10% NCS (*v*/*v*) and 1% penicillin/streptomycin (*v*/*v*). The cells were seeded at a density of 6000 viable cells/cm^2^ and differentiated in adipocytes according to Zebisch et al. [29]. Briefly, 3T3-L1 cells were seeded (day 1) and differentiation stimulated by incubation after 48 h with DMEM containing 10% FBS (*v*/*v*), 0.5 mM 3-isobutyl-1-methylxanthine (IBMX), 0.25 μM dexamethasone, 2 μM rosiglitazone and 1 μg/mL insulin (day 3). After 48 h, the medium was replaced with DMEM containing 10% (*v*/*v*) FBS and 1 μg/mL insulin (day 5). At days 7, 8 and 10, the media was replaced with 10% FBS-DMEM when 80–90% of the cells exhibited mature adipocyte phenotype. The cells were maintained at 37 °C in a 5% CO_2_ humidified atmosphere.

RAW 267.4 monocytes cells were cultured in DMEM supplemented with 1% penicillin/streptomycin (*v*/*v*) and 10% FBS (*v*/*v*). The cells were seed at 2.5 × 10^5^ in a six-well plate and the total volume was adjusted to 2 mL with growth medium and incubated to 24 h at 37 °C. After incubation, the cells were treated with the lipopolysaccharide (LPS) (1 µM) to 24 h and the media collected and designated as conditioned media (CM).

The efficiency of the cells growing in the presence of 1 mg/mL of DTP, digested albumin, digested glutelin and 100 µM of each pure peptide was assessed by aqueous solution CellTiter 96 one proliferation assay kit- MTS [3-(4,5-dimethylthiazol-2-yl)-5-(3-carboxymethoxyphenyl)-2-(4-sulfophenyl)-2H-tetrazolium, inner salt](Promega Corporation, Madison, WI, USA).

### 2.5. Experimental Design and Treatment of Cells with Digested Total Protein, Digested Albumin, Glutelin or Pure Peptides

Four experiments were conducted in order to analyze the effects of either DTP or digested albumin or digested glutelin or pure peptides on adipogenesis and inflammation on adipose tissue. A preventive (before) and inhibition (after) treatment approaches regarding exposure to induced inflammation by the addition of conditioned medium (CM) from inflamed macrophages:

#### 2.5.1. Experiment I: Effects of DTP or Digested Albumin or Glutelin in 3T3-L1 Adipocytes during the Differentiation Process.

The 3T3-L1 cells were seeded and differentiated as described above (Section 2.4) and the DTP or digested albumin or glutelin at 1 mg/mL were added to the culture media during the differentiation process (days 3, 5, 7, 8 and 10). A positive control, cells differentiated into adipocytes and not treated with the chia digested proteins, was included.

#### 2.5.2. Experiment II: Effects of Pure Peptides in 3T3-L1 Adipocytes during the Differentiation Process: 3T3-L1 Cells Were Seeded and Differentiated as Described above

The pure peptides NSPGPHDVALDQ (Pep1) and RMVLPEYELLYE (Pep2) at 100 µM were added to the culture media during the differentiation process (as described in Section 2.4), on days 3, 5, 7, 8 and 10. A positive control, cells differentiated into adipocytes and not treated with the chia digested proteins, was included.

#### 2.5.3. Experiment III: Effects of DTP or Digested Albumin or Glutelin before (Prevention) Induced Inflammation in Mature Adipocytes by Addition of Conditioned Media from Inflamed Macrophages

At 10th and 11th days of adipocyte differentiation (as described in Section 2.4), CM replaced 50% of grown media from inflamed macrophages together with DTP or digested albumin or glutelin at 1 mg/mL for 24 h. Untreated cells (negative control) were differentiated cells (mature adipocytes) but did not receive the conditioned media from macrophages or the digested proteins of chia. Positive control, differentiated cells (mature adipocytes) that received the conditioned media from macrophages but were not treated with the chia digested proteins.

#### 2.5.4. Experiment IV: Effects of DTP or Digested Albumin or Glutelin after (Inhibition) Induced Inflammation in Mature Adipocytes by Addition of Conditioned Media from Inflamed Macrophages (CM)

At 10th and 11th days of adipocyte differentiation (as described in Section 2.4), CM from inflamed macrophages replaced 50% of grown media and at days 12th and 13th DTP or digested albumin or glutelin at 1 mg/mL were added in addition to the grow media CM. Untreated cells (negative control) were differentiated cells (mature adipocytes) but did not receive the conditioned media from macrophages or the digested proteins of chia. Positive control, differentiated cells (mature adipocytes) that received the conditioned media from macrophages but were not treated with the chia digested proteins.

After each treatment, the growth medium and cell lysates were collected and frozen at −80 °C until analysis. All experiments were performed at least in duplicate.

### 2.6. Effect of Either Digested Total Protein, Digested Albumin or Digested Glutelin or Pure Peptides in Nitric Oxide and Reactive Oxygen Species Production

The accumulation of nitric oxide in the culture supernatant was measured by mixing 100 μL of the culture supernatant with the same volume of the Griess reagent and incubated at room temperature for 10 min. The absorbance was determined at 540 nm in a microplate reader (BioTek Instruments Inc., Winnoski, VT, USA) [30]. The concentration of nitrite was established from a standard curve of sodium nitrite (NaNO_2_) (y = 0.0087x + 0.0027, R^2^ = 0.99).

To analyze reactive oxygen species (ROS) production, the cells were seeded in dark 96 well plate in triplicate (as Section 2.4). At the last day of each experiment (Section 2.5), the media was replaced by H_2_DCFDA (2′,7′-dichlorodihydrofluorescein diacetate) in grown media (50 µM/total volume) and kept for 1 h in an incubator at 37 °C in 5% CO_2_. After this period, the plate was transferred to the microplate reader without washing and read with excitation wavelength at 485 nm and emission wavelength at 535 nm. Results were expressed as fluorescence intensity.

### 2.7. Effect of Either Digested Total Protein or Digested Albumin, Glutelin or Pure Peptides on Cellular Lipid Accumulation

The 3T3-L1 cells were seeded in 12-well plates and induced to differentiation. At the last day of each experiment (Section 2.5), the cells were fixed with 10% formalin, washed with 60% isopropanol and a working solution of Oil Red O was added to each well (0.35% *w*/*v* Oil Red O in isopropanol overnight). Then, pictures were taken and Oil Red O staining was eluted with 100% isopropanol for detection at 510 nm in a plate reader [31].

### 2.8. Impact of Either Digested Total Protein or Digested Albumin, Glutelin or Pure Peptides in PGE2, TNF-α, MCP-1 and Cytokines Secretion

Commercial kits were used to analyze prostaglandin 2 (PGE2) (500141) (Cayman Chemical), TNF-α (DY008), monocytes chemoattractant protein-1 (MCP-1) (DY479-05), interleukin-10 (IL-10) (DY417-05), IL-12 (DY419-05) and IL-6 (DY406-05) (R&D Systems), following the manufacturer’s instructions and according Grancieri, Martino and Gonzalez de Mejia [25]. The cell culture supernatants were diluted 1:5 (*v*/*v*, sample: buffer) for experiments I and II and 1:25 (*v*/*v*, sample: buffer) for experiments III and IV. The amount of PGE2 (y = −0.2766x + 0.3636, R^2^ = 0.99), TNF-α (y = 0.7991x − 2.0792, R^2^ = 0.99), MCP-1 (y = 0.7074x − 1.5536, R^2^ = 0.98), IL-6 (y = 0.7681x − 2.4798, R^2^ = 0.99); IL-10 (y = 0.7159x − 1.2982, R^2^ = 0.99), IL-12 (y = 0.7433x − 1.8984, R^2^ = 0.99) were calculated using log_10,_ including their respective standard curves that were run at the same time as treatments. Absorbance was determined at 450 nm and results were expressed in pg/mL.

### 2.9. Influence of Either Digested Total Protein or Digested Albumin, Glutelin or Pure Peptides in the Expression of Proteins Related to Adipogenesis and Inflammation Processes

The expression of proteins related with the adipogenesis and inflammation was measured by western blotting and according Grancieri, Martino and Gonzalez de Mejia [25]. Cell culture were collected after each treatment and lysed with RIPA lysis buffer, sonicated and added with Laemmli buffer (Bio-Rad) containing 5% β-mercaptoethanol, then the cell lysed were frozen at −80 °C. Protein concentration was quantified using RC-DC Assay (Bio-Rad) and 20 µg loaded in 4–20% Tris−HCl gels (Bio-Rad) for protein separation. Proteins were transferred to a PVDF membrane (polyvinylidene difluoride membrane, Hybond-P, Millipore, Billerica, MA, USA) and incubated overnight with respective primary antibodies (1:500) (COX-2, p65-NF, PPARγ, FAS, LPL or SREBP1) at 4 °C. The membranes were incubated with secondary antibody for 2 h (if required) and the protein bands visualized with a GL 4000 Pro Imaging system (Carestream Health Inc., Rochester, NY, USA). Band intensity was normalized using GAPDH antibody. All analyses were performed in duplicate and results expressed in % of expression compared to positive control.

### 2.10. Effects of Either Digested Total Protein or Digested Albumin, Glutelin or Pure Peptides on Inhibition of Lipase Activity and Triglyceride Content

3T3-L1 cells were cultured and treated as in Section 2.4 and Section 2.5. On the last day of each experiment, the cells were collected with RIPA buffer and stored at −80 °C. Inhibition of lipase activity was determined using a lipase activity assay kit (Cayman Chemical, Item No. 700640), following manufacturer’s instructions. Results were expressed as nmol/min/mL. Triglyceride content was analyzed using a triglyceride colorimetric assay kit (Cayman Chemical Item No. 10010303) according to manufacturer’s instructions. Results were expressed as mg/dL.

### 2.11. Potential Inhibitory Interactions of PPARγ, FAS and MAGL by Peptides from Digested Total Protein, Digested Albumin or Glutelin: In Silico Analyses

Interactions of generated peptides from DTP and digested albumin and glutelin, including Pep1 and Pep2, with PPARγ, FAS and monoacylglycerol lipase (MAGL) were evaluated by in silico analysis. The peptides were designed using Instant MarvinSketch Software (ChemAxon Ltd., Boston, MA, USA). The crystal structure files of PPARγ, FAS and MAGL were obtained from the Protein Data Bank (PDB: 5DSH, 2PX6 and 3PE6 respectively). Flexible torsions, charges and grid size were assigned by AutoDock Tools and docking calculations were performed using AutoDock Vina [32]. The binding pose with the lowest binding energy (highest binding affinity) was selected as a representative image to be visualized in the computer program Discovery Studio version 2016 Client (Dassault Systèmes BIOVIA, San Diego, CA, USA).

Moreover, the pharmacological controls GW9962 (2-chloro-5-nitro-*N*-phenylbenzamide) [33], Orlistat [34] and endocannabinoid 2-arachidonoylglycerol (2-AG) [35] were used, respectively for PPARγ, FAS and MAGL.

### 2.12. Statistical Analysis

Results were expressed as the mean ± standard deviation (SD) and analyzed by *one-way* analysis of variance (ANOVA) and post hoc Tukey test. Differences were considered significant at *p* < 0.05. The western-blot analysis were performed in duplicate and all the other analysis were performed in triplicate, from at least two biological independent replicates, using GraphPad Prism 7.

## 3. Results

### 3.1. Effect of Digested Total Protein (DTP), Digested Albumin and Glutelin and Pure Peptides on Adipogenesis Development in the Adipocytes Cells

#### 3.1.1. The Digested Total Protein (DTP), Digested Albumin and Glutelin and Pure Peptide Pep2 Reduced the Differentiation of Adipocytes and Oxidative Stress in the Adipogenesis Development

**Figure 1:**
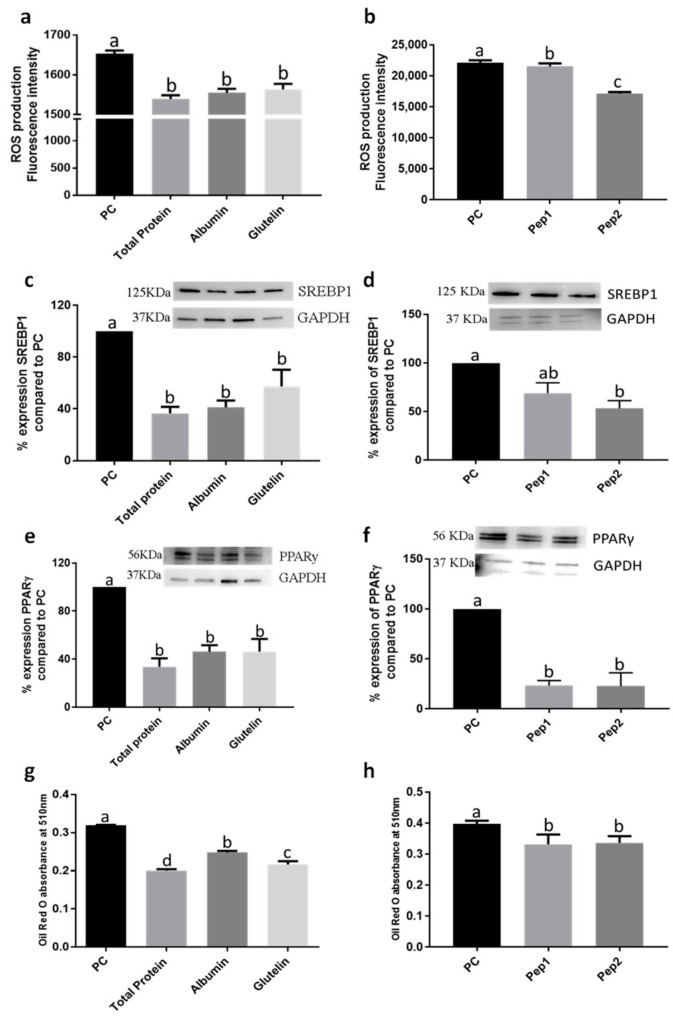
Effect of digested total protein, digested albumin or glutelin from chia seeds and pure peptides NSPGPHDVALDQ (Pep1) and RMVLPEYELLYE (Pep2) from glutelin to prevent the adipogenesis process. Reactive oxygen species (ROS) production (**a**,**b**); SREBP-1 (**c**,**d**) expression; PPARγ expression (**e**,**f**); Oil Red-O absorbance (**g**,**h**). The proteins were added during the differentiation period (days 3, 5, 7, 8 and 10) and PC (positive control) received no treatment. All experiments were performed in at least two independent trials run with triplicate data points. Different letter per column means statistically different among the proteins by ANOVA and post-hoc Tukey-test (*p* < 0.05). ROS: Reactive oxygen species; SREBP-1: sterol regulatory element-binding protein 1; PPARγ: peroxisome proliferator-activated receptor gamma; GAPDH: glyceraldehyde 3-phosphate; PC: Positive control.

Digested total protein (DTP), digested albumin and glutelin (Figure S1) and pure peptides showed a high cell viability, demonstrating that these samples do not interfere with cell growth and normal cell metabolism (Figure S2). Furthermore, these samples reduced ROS production (*p* < 0.05) (Figure 1a,b). DTP, digested albumin and glutelin and Pep2 (pure peptide 2: RMVLPEYELLYE) reduced expression of sterol regulatory element-binding protein 1 (SREBP1) by −63.4, −58.8, −53.3 and −46.7%, respectively (*p* < 0.05) (Figure 1c,d). The expression of PPARγ was reduced by DTP, digested albumin and glutelin and pure peptides, primarily by Pep2 (−77.1%; *p* < 0.05) (Figure 1e,f).

#### 3.1.2. The Digested Total Protein (DTP), Digested Albumin and Glutelin and Pure Peptides, Pep1 and Pep2, Reduced the Lipid Accumulation in the Adipogenesis Development

**Figure 2:**
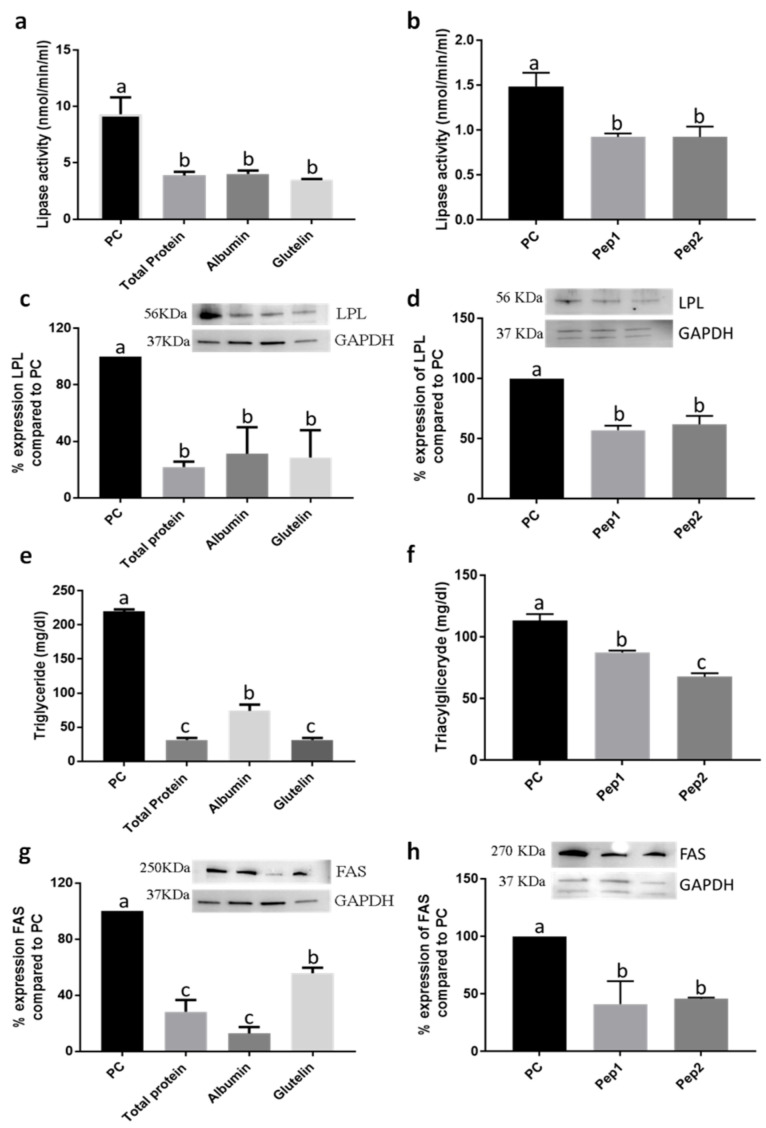
Effect of digested total protein, digested albumin or glutelin from chia seeds and pure peptides NSPGPHDVALDQ (Pep1) and RMVLPEYELLYE (Pep2) from glutelin of chia seeds to prevent the adipogenesis process. Lipase activity (**a**,**b**); LPL expression (**c**,**d**); triglyceride content (**e**,**f**); FAS expression (**g**,**h**). Treatments were added during the differentiation period (days 3, 5, 7, 8 and 10) and PC received no treatment. All experiments were performed in at least two independent trials run with triplicate data points. Different letter per column means statistically different among the proteins by ANOVA and post-hoc Tukey-test (*p* < 0.05). LPL: lipase lipoprotein; FAS: fatty acid synthase; GAPDH: glyceraldehyde 3-phosphate; PC: Positive control.

Every treatment with DTP, digested albumin and glutelin and pure peptides, Pep1 (pure peptide 1: NSPGPHDVALDQ) and Pep2, reduced lipid accumulation (Figure 1g,h and Figure S3), especially by DTP (−37.2%; *p* < 0.05). Moreover, the activity of lipase was also reduced by −58.3%, −56.9% and −62.6% by DTP, digested albumin and glutelin, respectively (*p* < 0.05) (Figure 2a). However, reduction by pure peptides was −37.8%, in comparison to the positive control (Figure 2b). Every sample reduced (*p* < 0.05) lipase lipoprotein (LPL) expression (Figure 2c,d), triglyceride content (Figure 2e,f) and fatty acid synthase (FAS) expression (Figure 2g,h). DTP reduced the content of triglycerides and FAS expression by −85.7% and −71.6%, respectively (*p* < 0.05).

#### 3.1.3. The Digested Albumin and Glutelin and Pure Peptides, Pep1 and Pep2, Reduced the Inflammatory Markers in the Adipogenesis Development

**Figure 3:**
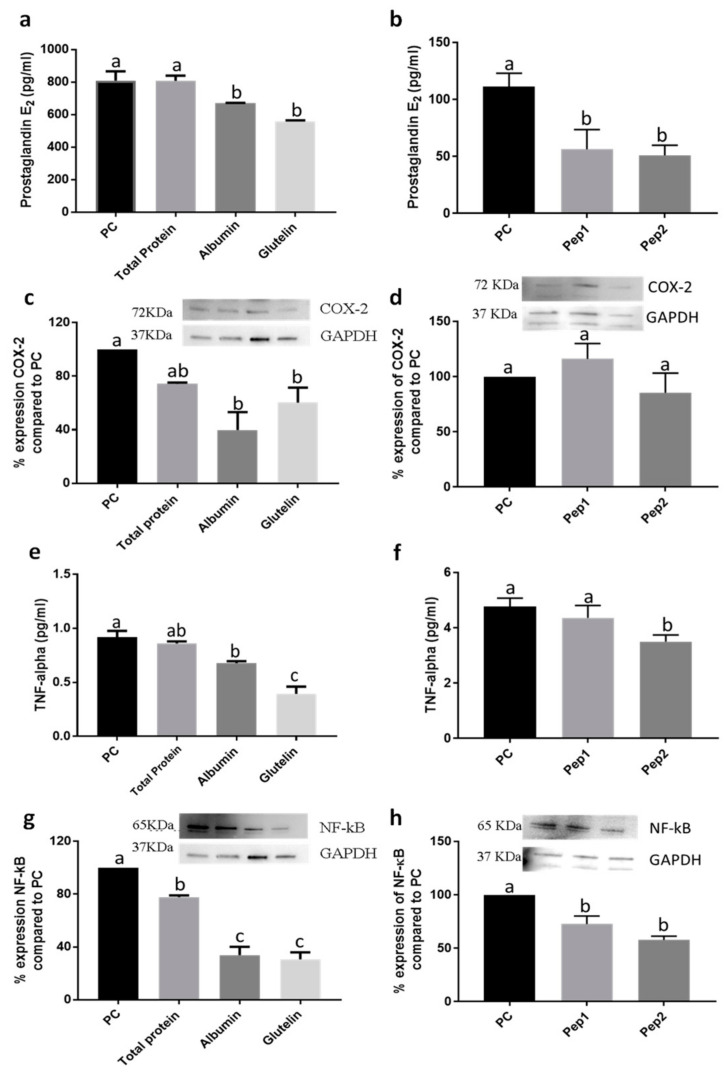
Effect of digested total protein, digested albumin, digested glutelin from chia seeds and pure peptides NSPGPHDVALDQ (Pep1) and RMVLPEYELLYE (Pep2) from glutelin of chia seeds, in inflammatory markers to prevent the adipogenesis process. PGE2 secretion (**a**,**b**); COX-2 expression (**c**,**d**); TNF-α secretion (**e**,**f**); NF-κB expression (**g**,**h**). Treatments were added during the differentiation period (days 3, 5, 7, 8 and 10) and the PC received no treatment. All experiments were performed in at least two independent trials run with triplicate data points. Different letter per column means statistically different among the proteins by ANOVA and post-hoc Tukey-test (*p* < 0.05). PGE2: prostaglandin 2; COX-2: cyclooxygenase2; TNF-α: Tumor necrosis factor alpha; NF-kB: Factor nuclear kappa B; PC: Positive control.

Digested albumin and glutelin but not DTP, were effective on reducing the secretion of prostaglandin-2 (PGE2) (*p* < 0.05) (Figure 3a). Pure peptides Pep1 and Pep2 reduced PGE2 secretion by −49.3 and −54.1%, respectively (*p* < 0.05) (Figure 3b). Digested albumin and glutelin also reduced the expression of cyclooxygenase-2 (COX-2) in −68.1 and −48.1%, respectively (*p* < 0.05). However, DTP and pure peptides had no effect (*p <* 0.05) (Figure 3c,d). The secretion of tumor necrosis factor alpha (TNF-α) was reduced by digested albumin, glutelin and Pep2 (*p* < 0.05) (Figure 3e,f); all proteins reduced NF-κB expression, particularly glutelin (−69.3%; *p* < 0.05) (Figure 3g,h). Glutelin, Pep1 and Pep2 reduced the secretion of IL-6 in −28.4%, −18.9% and −33.8%, respectively, as compared with PC (positive control) (*p* < 0.05) (Table S1). In addition, pure peptides decrease the IL-10 secretion (Table S1) (*p* < 0.05). IL-12 secretion (Table S1) and nitric oxide (NO) production (data not shown) were not statistically different to PC (*p >* 0.05).

### 3.2. Effect of DTP and Digested Albumin and Glutelin on Inflammation in Mature Adipocytes

#### 3.2.1. DTP and Digested Albumin and Glutelin Fractions Reduced the Oxidative Stress in the Prevention and Inhibition of Induced Inflammation in Mature Adipocytes

**Figure 4:**
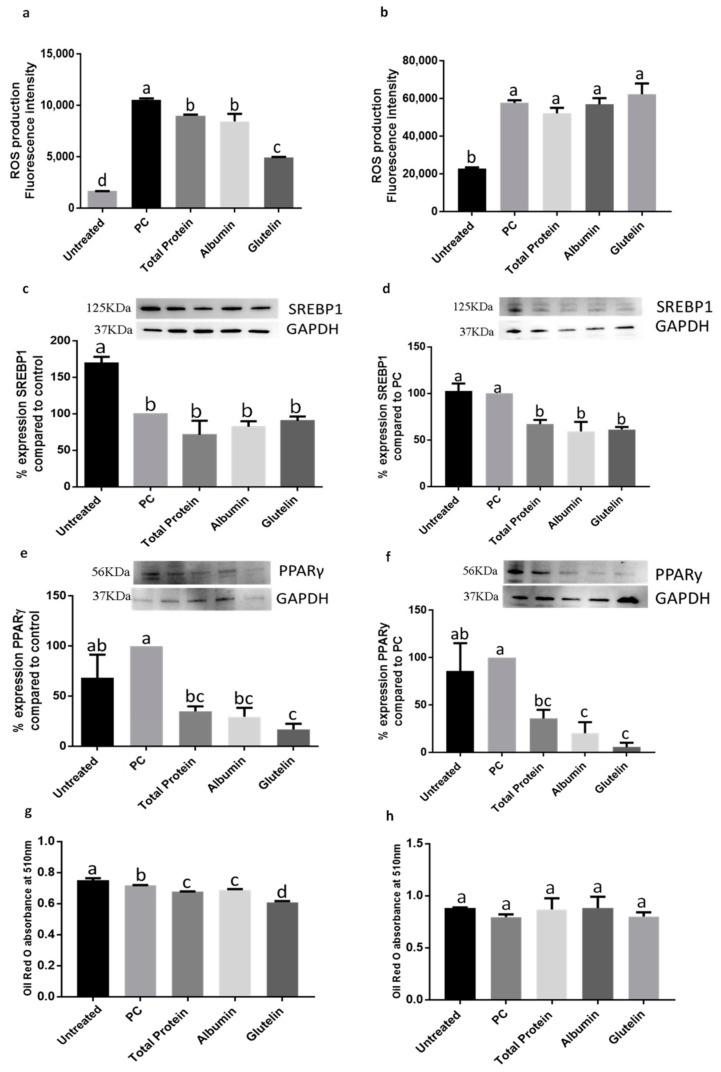
Effect of digested total protein and digested albumin and glutelin from chia seeds to prevent and inhibit the oxidative stress and markers of adipogenesis process in mature adipocytes stimulated by inflamed macrophages. ROS production on prevention (**a**) and inhibition (**b**); SREBP-1 expression on prevention (**c**) and inhibition (**d**); PPARγ expression on prevention (**e**) and inhibition (**f**); Oil Red O absorbance on prevention (**g**) and inhibition (**h**). Untreated group receive any treatment, PC receive only the CM. All experiments were performed in at least two independent trials run with triplicate data points. Different letter per column means statistically different among the proteins by ANOVA and post-hoc Tukey-test (*p* < 0.05). CM: conditioned media from inflamed macrophages; ROS: Reactive oxygen species; SREBP-1: sterol regulatory element-binding protein 1; PPARγ: peroxisome proliferator-activated receptor gamma; GAPDH: glyceraldehyde 3-phosphate; PC: Positive control.

ROS production was reduced when DTP or digested albumin and glutelin were added to prevent the development of inflammation (*p* < 0.05). Glutelin, specially, reduced ROS production −53.3% (*p* < 0.05) (Figure 4a). However, when proteins were used to inhibit already existing inflammation, there was not effect (*p >* 0.05) (Figure 4b).

#### 3.2.2. DTP and Digested Albumin and Glutelin Fractions Reduced Lipid Accumulation in the Prevention and Inhibition of Induced Inflammation in Mature Adipocytes

The expression of SREBP-1 was not reduced by preventing inflammation (*p* > 0.05) (Figure 4c) but on inhibition, DTP and digested albumin and glutelin reduced the expression of this protein in −33.0%, −40.8% and −38.7%, respectively (*p* < 0.05) (Figure 4d). The use of DTP and digested albumin and glutelin in both, prevention and inhibition of induced inflammation, decreased the expression of PPARγ (*p* < 0.05). Glutelin reduced PPARγ expression in −83.2% and −94.2% in prevention and inhibition of induced inflammation, respectively (*p* < 0.05) (Figure 4e,f). The Oil red-O accumulation was reduced on prevention experimental approach by all proteins (*p* < 0.05) (Figure 4g) but not for the inhibition approach (*p* > 0.05) (Figure 4h).

**Figure 5:**
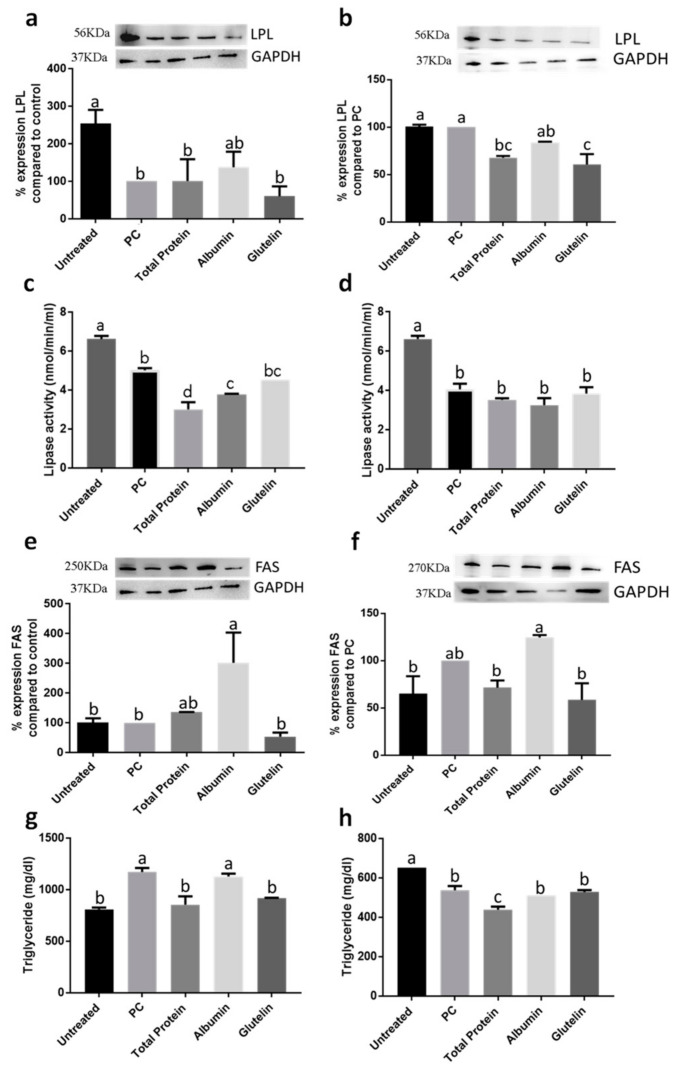
Effect of digested total protein and digested albumin and glutelin from chia seeds to prevent and inhibit the adipogenesis process markers in mature adipocytes stimulated by inflamed macrophages. LPL expression on prevention (**a**) and inhibition (**b**); LPL activity on prevention (**c**) and inhibition (**d**); FAS expression on prevention (**e**) and inhibition (**f**); triglyceride content on prevention (**g**) and inhibition (**h**). Untreated receive any treatment, PC receive only the CM. All experiments were performed in at least two independent trials run with triplicate data points. Different letter per column means statistically different among the proteins by ANOVA and post-hoc Tukey-test (*p* < 0.05). CM: conditioned media; LPL: lipase lipoprotein; FAS: fatty acid synthase; GAPDH: glyceraldehyde 3-phosphate; PC: Positive control.

Digested albumin increased the expression of LPL similar to untreated cells (Figure 5a) in prevention of inflammation (Figure 5a); on inhibition, DTP and glutelin reduced the expression of LPL (−32.2% and −39.3%, respectively; *p* < 0.05) (Figure 5b). On prevention, DTP and albumin reduced lipase activity (−40.1% and −24.6%, respectively; *p* < 0.05) (Figure 5c); however, the activity of lipase was not changed by any treatment in inhibition approach (*p* > 0.05) (Figure 5d). Digested albumin increased FAS expression in both treatment approaches, prevention (Figure 5e) and inhibition (Figure 5f) of induced inflammation (*p* < 0.05). Furthermore, glutelin reduced triacylglyceride content by −21.8% (Figure 5g) when preventing inflammation and DTP reduced triglyceride content also in both, prevention (Figure 5g) and inhibition of induced inflammation (*p* < 0.05) (Figure 5h).

#### 3.2.3. DTP and Digested Albumin and Glutelin Reduced Inflammatory Markers in Prevention and Inhibition of Induced Inflammation

**Figure 6:**
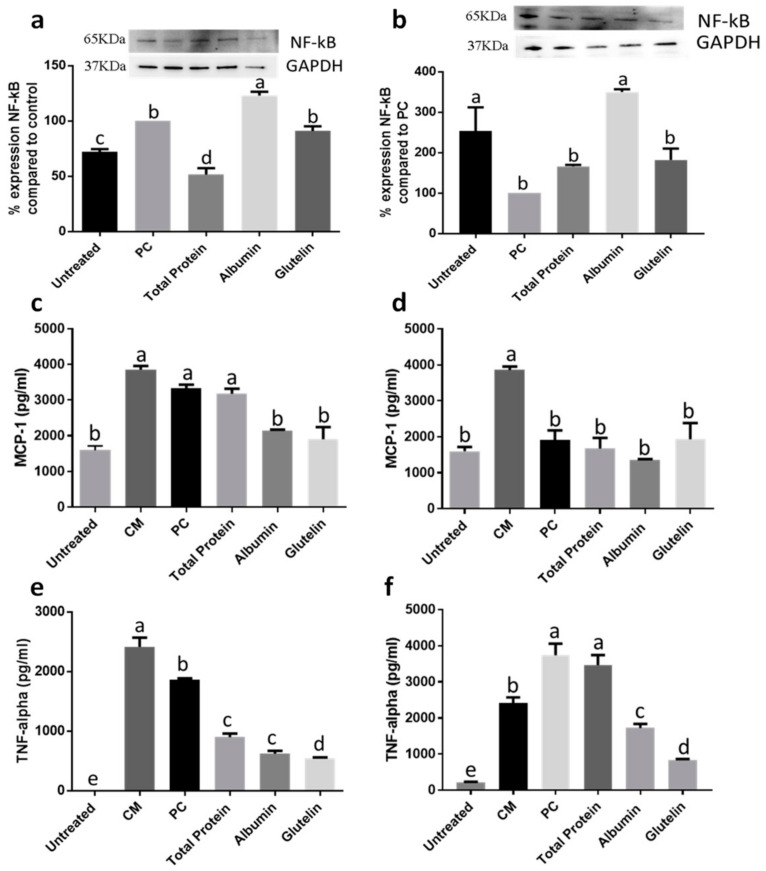
Effect of digested total protein and digested albumin and glutelin from chia seeds to prevent and inhibit the inflammation in mature adipocytes stimulated by inflamed macrophages. NF-κB expression on prevention (**a**) and inhibition (**b**); MCP-1 secretion on prevention (**c**) and inhibition (**d**); TNF-α secretion on prevention (**e**) and inhibition (**f**). Untreated receive any treatment, PC receive only the CM. All experiments were performed in at least two independent trials run with triplicate data points. Different letter per column means statistically different among the proteins by ANOVA and post-hoc Tukey-test (*p* < 0.05). CM: conditioned media; NF-kB: Factor nuclear kappa B; GAPDH: glyceraldehyde 3-phosphate; MCP-1: monocyte chemoattractant protein 1; TNF-α: Tumor necrosis factor alpha; PC: Positive control.

NF-κB expression was reduced only by DTP in the prevention treatment approach (−48.4%; *p* < 0.05) (Figure 6a). During inhibition, any sample showed effect for this marker (*p* > 0.05) (Figure 6b). The monocyte chemoattractant protein-1 (MCP-1) secretion was reduced −35.8% and −42.8%, respectively by digested albumin and glutelin during the prevention process (*p* < 0.05) (Figure 6c). However, any protein reduced MCP-1 during inhibition of induced inflammation (*p >* 0.05) (Figure 6d). Every protein reduced TNF-α secretion in prevention (−51.6% to DTP, −66.6% to digested albumin and −70.9% to digested glutelin; *p* < 0.05) (Figure 6e) and only digested albumin and glutelin reduced the secretion of this cytokine on inhibition of already produced inflammation (−53.8 to digested albumin and −77.9% to digested glutelin; *p* < 0.05) (Figure 6f). 

**Figure 7:**
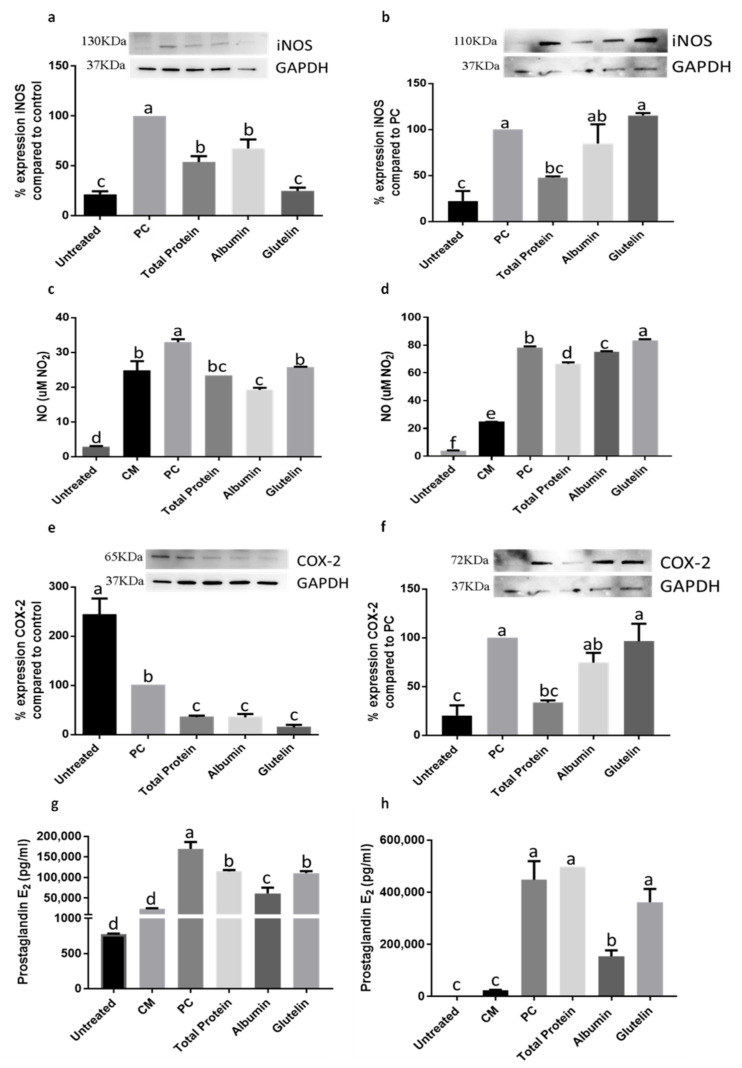
Effect of digested total protein and digested albumin and glutelin from chia seeds to prevent and inhibit the inflammation in mature adipocytes stimulated by inflamed macrophages. iNOS expression on prevention (**a**) and inhibition (**b**); NO secretion on prevention (**c**) and inhibition (**d**); COX-2 expression on prevention (**e**) and inhibition (**f**); PGE2 secretion on prevention (**g**) and inhibition (**h**). Untreated receive any treatment, PC receive only the CM. All experiments were performed in at least two independent trials run with triplicate data points. Different letter per column means statistically different among the proteins by ANOVA and post-hoc Tukey-test (*p* < 0.05). CM: conditioned media; iNOS: inducible nitric oxide synthase; GAPDH: glyceraldehyde 3-phosphate; NO: nitric oxide; COX-2: cyclooxygenase2; PGE2: prostaglandin 2; PC: Positive control.

In preventing the inflammation process, DTP and digested albumin and glutelin reduced inducible nitric oxide synthase (iNOS) expression, mainly digested glutelin which reduced (−75%) the expression of iNOS (*p* < 0.05) (Figure 7a). Only DTP reduced iNOS expression (−52.1%) in the inhibition of induced inflammation (*p* < 0.05) (Figure 7b). NO secretion was reduced by every protein in prevention of inflammation (*p* < 0.05) (Figure 7c). However, on inhibition just DTP and digested albumin showed effect (*p* < 0.05) (Figure 7d). The COX-2 expression was reduced by every protein in prevention (−64.5%, DTP; −64.8%, digested albumin; −85.3%, digested glutelin; *p* < 0.05) (Figure 7e) and just by DTP (−66.4%) on inhibition of inflammation (*p* < 0.05) (Figure 7f). Secretion of PGE2 was also reduced by every sample in the prevention process (*p* < 0.05) (Figure 7g) and digested albumin reduced PGE2 secretion (−65.5%) on inhibition (*p* < 0.05) (Figure 7h). The secretion of cytokines had no changes in comparison with the PC, in both, prevention and inhibition of induced inflammation (*p >* 0.05) (Table S1). 

### 3.3. In Silico Interaction of Peptides from Digested Albumin and Glutelin and Pure Peptides with PPARγ, FAS and MAGL

**Figure 8:**
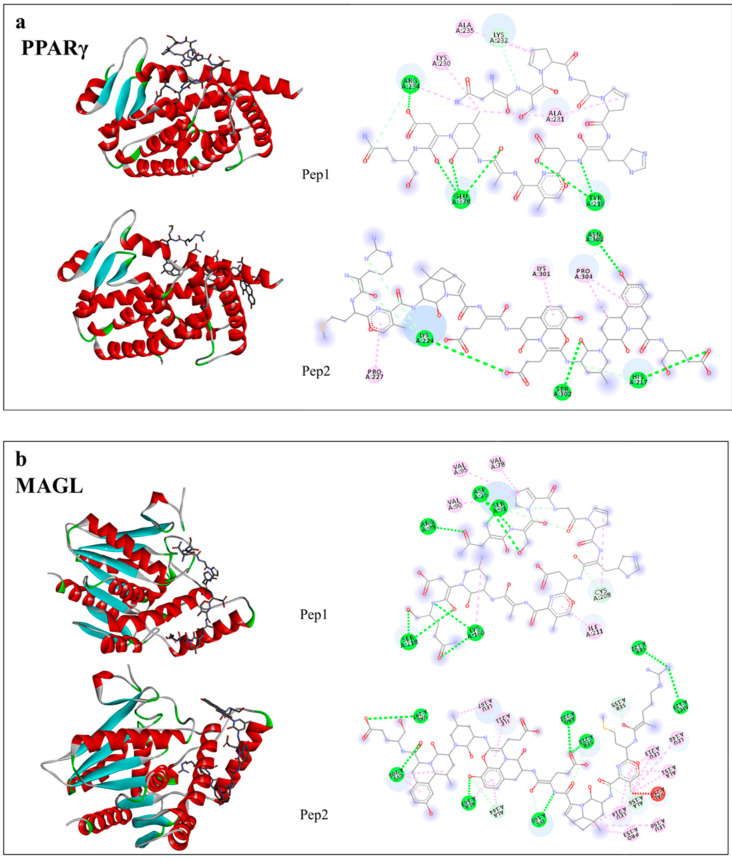

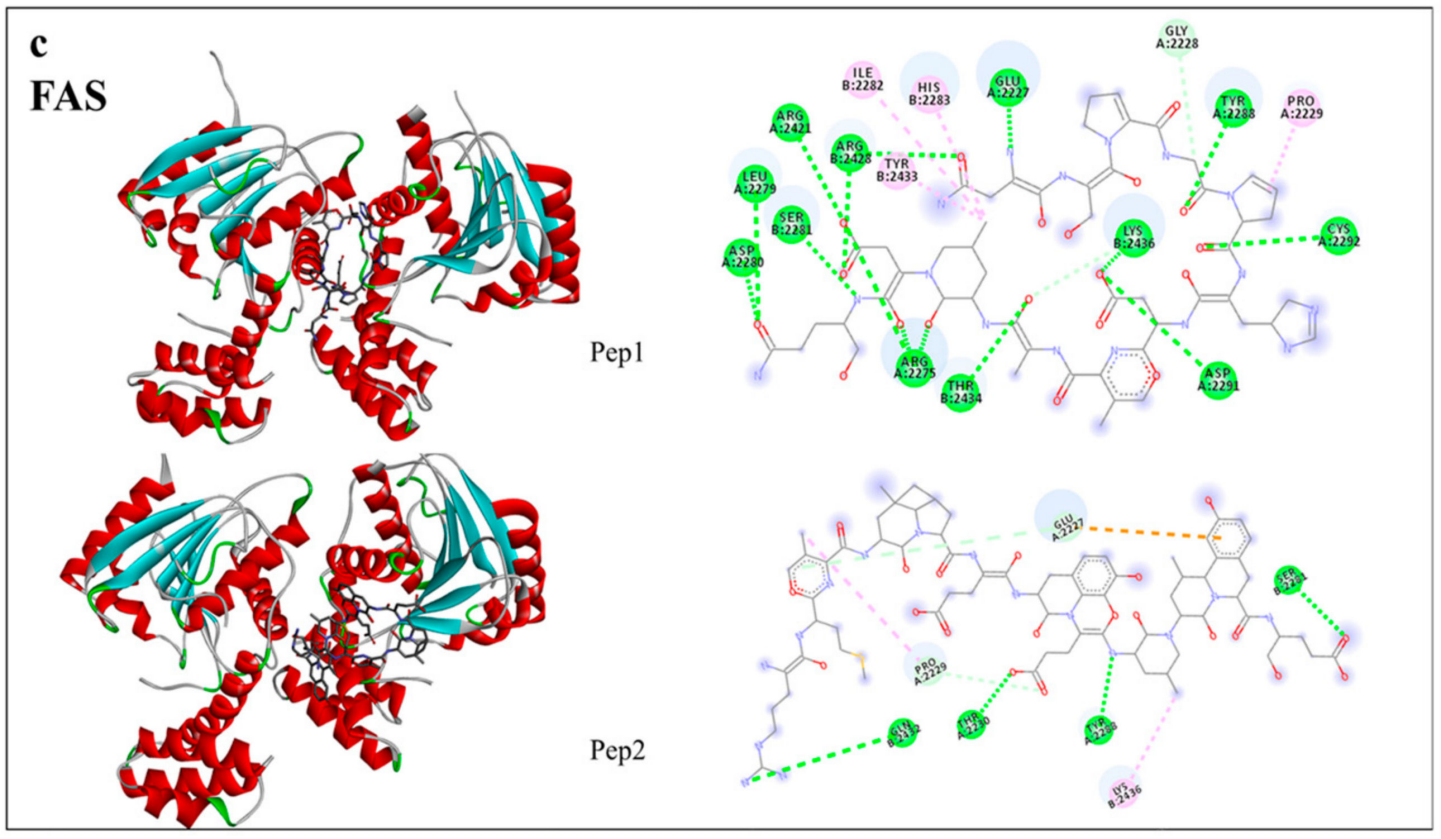
The in silico interaction of the peptides NSPGPHDVALDQ (Pep1) and RMVLPEYELLYE (Pep2) found in digested glutelin, with PPARγ (**a**) MAGL (**b**) and FAS (**c**). These analyzes were performed by AutoDock Vina^®^ and visualized by Discovery Studio 2016 Client^®^.

Peptides NSPGPHDVALDQ (Pep1) and RMVLPEYELLYE (Pep2) showed interactions with enzymes FAS and monoacylglycerol lipase (MAGL) and PPARγ receptor, related to the adipogenesis process. Pep2 showed the highest interaction with PPARγ by lowering the estimated free energy (EFE) (−6.9 kcal/mol) (Figure 8a) and with MAGL by also lowering EFE (−7.3 kcal/mol) (Figure 8b). However, Pep1 had the highest interaction with FAS (−7.3 kcal/mol) (Figure 8c). Other identified peptides in chia seed, peptides TGPSPTAGPPAPGGGTH and YLGAHPGTAN, both from digested albumin, showed the highest interaction with PPARγ (−9.1 kcal/mol) and MAGL (−7.8 kcal/mol), respectively. The peptide APSPPVLGPP from DTP, showed the highest interaction with FAS (−9.8 kcal/mol). These values were lower (more interaction) than the pharmacological controls tested, which showed EFE: −8.5, −7.4 and −5.5 kcal/mol, respectively for PPARγ, FAS and MAGL (Table S2). 

## 4. Discussion

This study shows in vitro the effects of digested proteins from chia seed, including total protein and its fractions, albumin and glutelin, on adipogenesis and inflammation of adipose tissue, markers of obesity and its complications. This investigation shows that protein fractions, generated by digestion of chia proteins and pure peptides can prevent adipogenesis by reducing markers related with this process and on the development of in vitro induced inflammation of adipose tissue. Although no study has evaluated the digested proteins and their peptides from chia seeds on markers related to obesity, some studies evaluated the whole chia seed in animal and clinical trials and found promising results against obesity [36].

In this research, we performed the cell viability test with the independent samples (digested proteins or pure peptides) before treatment to verify if these samples could interfere with normal cell growth and development. Then, we observed that the digested proteins and peptides did not interfere with the cell growth or the normal cell metabolism. In addition, we maintained the negative and/or positive controls that allowed us to verify the behavior of the cells with and without treatments. Therefore, we can affirm that the effects we found of the samples on the markers of adipogenesis and inflammation were caused by the digested proteins or the isolated peptides. Therefore, the peptide preparations did not directly interfere with the performance of the employed enzymatic and immunological assays. The peptide preparations were successful at decreasing the markers of inflammation.

In obesity, adipose tissue undergoes expansion, which may ultimately compromise its function [5]. Mainly PPARγ, considered as the ‘master regulator’ of adipogenesis, controls this expansion and its expression is sufficient to induce adipocyte differentiation. Furthermore, PPARγ is required for maintenance of this differentiated state [4,31]. A recent study demonstrated that adipocytes PPARγ knockout did not develop adipogenesis, demonstrating the role of this marker in the development of obesity and its complications, such as insulin resistance. In this study, the authors also used GAPDH as an internal control to check the expression of PPAR and other genes related to this pathway [37]. In the present research, DTP, digested albumin and glutelin and pure peptides, reduced the expression of PPARγ during the adipogenesis process, preventing the formation of adipocytes. In addition, DTP reduced the expression of PPARγ in mature adipocytes exposed to media from inflamed microphages, showing their efficacy to reverse the adipogenesis process. This ability of peptides against PPARγ was confirmed by in silico analysis, which peptides had more interaction than the pharmacological control used (GW9962).

The PPARγ expression can be stimulated by other factors such as the SREBP1 [38]. The expression of this protein was reduced, in the present research, when DTP, digested albumin and glutelin or Pep2 were added to the cells during adipogenesis development. SREBP1 also mediates the induction of lipogenesis by insulin in adipocytes, together with PPARγ, the expression of proteins, such as adipocyte fatty acid-binding protein (aP2), FAS, LPL [4,39]. LPL is a key enzyme in lipid transport and metabolism and plays a crucial role in human lipid homeostasis and energy balance. LPL is responsible for catalyzing lipolysis of triglycerides in lipoproteins, as very low-density lipoproteins (VLDL), providing free fatty acids (FFAs) which are used for lipid storage, suggesting a role for LPL in initiation and development of obesity, becoming a marker for adipocyte differentiation [40].

In this study, LPL activity and expression were reduced when DTP, digested albumin and glutelin and pure peptides where added during adipogenesis and with DTP treatment in mature inflamed adipocytes. These results show the effectivity of the treatments, especially DTP, to reduce markers of adipogenesis. The content of triacylglycerides and Oil Red O, a differentiation parameter [31], was reduced drastically by every treatment when DTP, digested albumin and glutelin or pure peptides were added during the adipogenesis process and by DTP and digested glutelin in prevention of inflammation. However, digests from albumin increased the expression of LPL and the levels of triacylglycerides. This is probably due to inflammation, mainly the presence of TNF-α, which blocks transcription of LPL [41]. Reduction of obesity was observed in overweight and diabetics adults that consumed chia seeds during 24 weeks [42] and for overweight and obesity adults during 12 weeks [43]. Furthermore, rats fed with chia seeds reduced the visceral adiposity index and decreased the retroperitoneal and omental fat depositions [44]. These effects can be associated with the antiadipogenic and antilipogenic effects of digested chia proteins observed in the present study.

Another marker stimulated by SREBP1 and PPARγ is FAS. This enzyme catalyzes de novo synthesis of fatty acids by production of palmitate from malonyl-CoA and acetyl-CoA. Palmitate is subsequently used as the precursor for the synthesis of complex lipid molecules that can be used for energy storage, membrane assembly and repair and secretion in the form of lipoprotein triglycerides [45,46]. Moreover, inhibition of FAS activity may block adipocyte differentiation and reduce adipogenesis [47]. In this research, every chia sample tested was able to reduce FAS expression on preventing adipogenesis and the peptides from chia seed had highest interaction by in silico analysis than Orlistat, its pharmacological control. Wistar rats fed a high sucrose diet and treated with chia seeds, during 3 weeks or 5 months, reduced SREBP-1 expression, plasmatic triacylglycerol, liver triacylglycerol and FAS activity [48].

Furthermore, the expansion of adipose tissue, both in number of cells (hyperplasia) and in size due to the deposition of fat (hypertrophy), induces the expansion of adipose tissue, caused by adipogenesis. In addition, lead to increased secretion of pro-inflammatory cytokines and chemokines by adipocytes, such as TNF-α, IL-6, MCP-1, adiponectin, resistin, among others. These molecules increase the local inflammation and stimulate monocytes into adipose tissue. This condition activates their differentiation to macrophages that begin to secrete pro-inflammatory cytokines and chemokines that drive to a chronic low-grade inflammatory state with systemic effects. Consequently, the damages caused by obesity on human organism, mainly induction of diseases, such as diabetes, cancer and cardiovascular diseases. [31,39,49] This process may lead to activation of NF-κB and downstream pro-inflammatory genes involving TNF-α, IL-6 and MCP-1 and others [50]. In this study, we observed that the reduction of adipogenesis contributed to the reduction of inflammation given the reduction of the expression of NF-κB by DTP, digested albumin and glutelin and pure peptides when added during the adipogenesis process. In mature adipocytes, NF-κB had its expression reduced only by DTP. Despite results observed regarding NF-κB expression, digested albumin, glutelin and Pep2 decreased TNF-α secretion when preventing adipogenesis. However, secretion of TNF-α was reduced by every treatment, while MCP-1 only by digested albumin and glutelin when preventing inflammation. When adipocytes received the digests from proteins after inflammation was established, only TNF-α had its expression reduced by digested albumin and glutelin. In addition, digested glutelin and pure peptides reduced IL-6 secretion when preventing adipogenesis and this was the only effect observed regarding cytokines in all experiments. The cytokines are regulated not only by NF-κB but also the janus kinase (JAK), signal transducer and activator of transcription (STAT), mitogen-activated protein kinase (MAPK) and the phosphatidylinositol-3′-kinases (PI3K) pathways. These have been associated with the production of cytokines [51] and may have regulated the secretion of these small molecules in our study. Previous work from our group showed that peptides from chia seed had in silico interaction with p65-NF-κB, mainly the peptide HYGGPPGGCR, from DTP (EFE: −7.1 kcal/mol), which had the highest interaction with this inflammatory marker [25]. These data help to explain why only DTP reduced the expression of NF-κB in adipocytes. In other studies, the reduction of PPARγ expression/activity was associated with reduced inflammation in adipose tissue [31,39], as observed in this study. Further, the results can help to explain results with animals [52] and humans [17,18,43,17] that have found beneficial effects of chia seed against obesity and its complications.

Another enzyme related with inflammation is COX-2, which catalyzes the conversion of arachidonic acid to prostaglandin H2, a precursor of other inflammation mediators, as PGE2 [53]. This event was observed when the adipocytes received digested albumin and glutelin to prevent adipogenesis and with every sample when preventing inflammation. In this case, both digested proteins reduced COX-2 expression and PGE2 secretion. However, pure peptides did not change the COX-2 expression but reduced PGE2 secretion on preventing adipogenesis. These events were perhaps due to modulation by other proteins related with the COX-2 pathway, as p38 mitogen-activated protein kinase (p38MAPK) [54] and cycle AMP-protein kinase-A-kinase anchor proteins (cAMP-PKA-AKAP) pathway [55].

iNOS is another enzyme related to inflammation [56] that was evaluated in this research. Obesity increases expression of iNOS, which catalyzes NO synthesis and contributes to metabolic deregulation in adipocytes as well as stimulation of PPARγ expression [57]. In this investigation, iNOS expression and NO secretion were reduced by every treatment when preventing inflammation. In the inhibition of inflammation, just DTP reduced iNOS expression and NO secretion; also, albumin reduced NO levels. The effectiveness of chia seed to reduce plasma nitrite was observed in hypertensive and overweight adults that ingested 35 g/day of chia seeds [17].

Associated with the effectiveness of digested proteins on inflammation, every treatment reduced production of ROS on prevention of adipogenesis and inflammation. ROS can be generated into adipocytes from certain saturated free fatty acids (laurate, myristate and palmitate) [58] and by chronic stress, glucocorticoids, mineralocorticoids and angiotensin-II [59]. ROS can induce insulin resistance and recruitment of immune cells into adipose tissue, increasing adipose tissue inflammation [58]. In addition, ROS increases secretion of leptin, MCP-1, IL-6 and TNF-α by adipocytes and decreases adiponectin production [59]. These facts may explain the poor results of inhibition with digested proteins in inflamed adipocytes. Moreover, the effectiveness of complete chia seed has been observed by the increase of IL-10 [52], an anti-inflammatory cytokine, as well as the antioxidant capacity in rats [59,61].

**Figure 9:**
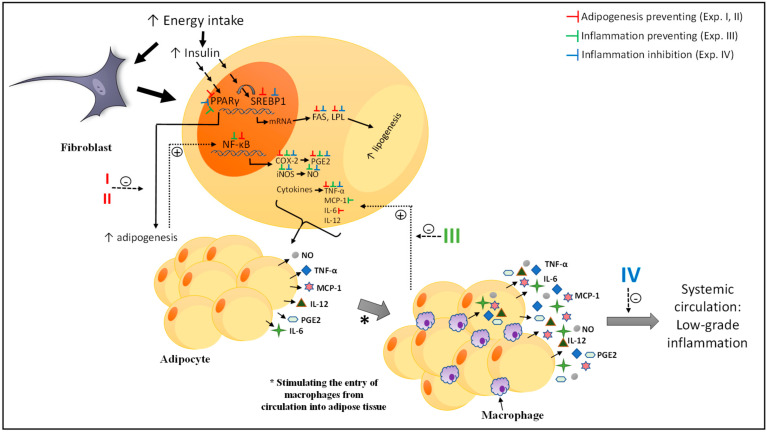
Proposed mechanism of the effect of DTP, digested albumin, digested glutelin or pure peptides in the prevention of adipogenesis (experiments I and II, the red symbols), in the prevention of inflammation (experiments III, the green symbols) and inhibition of establishing inflammation (experiments IV, the blue symbols). Red symbols: On prevention of adipogenesis, DTP, digested albumin, glutelin and pure peptides reduced the expression of PPARγ, SREBP1, FAS, lipoprotein lipase (LPL), nuclear factor-kappa B (NF-κB), cyclooxygenase-2 (COX-2), and inducible nitric oxide synthase (iNOS) and the secretion of PGE2, TNFα and albumin and pure peptides decrease the levels of IL-6. Green symbols: On prevention of inflammation, DTP, digested albumin and glutelin decreased the expression of PPARγ, COX-2 and iNOS and the NO, PGE2, TNFα secretion. DTP reduce the NF-κB expression. Digested albumin and glutelin reduced the MCP-1 secretion. Blue symbols: On inhibition of inflammation, DTP, digested albumin and glutelin decreased the expression of PPARγ and SREBP1. DTP reduce the expression of LPL, FAS, iNOS, COX-2, the triacylglycerol content, lipase activity, NO secretion. Digested albumin reduced the lipase activity and the secretion of NO, PGE2 and TNFα. Digested glutelin decreased the expression of LPL and FAS and TNFα secretion. DTP: digested total protein; PPARγ: peroxisome-proliferator-activated receptors gamma, SREBP1: sterol regulatory element-binding protein-1, FAS: fatty acid synthase, LPL: lipoprotein lipase, NF-κB: factor nuclear kappa B, COX-2: ciclooxigenase-2, iNOS: inducible nitric oxide synthase, MCP-1: Monocyte chemoattractant protein-1, NO: nitric oxide, PGE2: prostaglandin E2, IL-6: Interleukin 6.

It is important to highlight that the variability of the results was due to the biological nature and independence of the experiments. In other words, how each experiment was conducted independently; the variation between the experiment with digested protein and pure peptides (experiment I and II) or in the prevention and treatment (experiment III and IV) was due to the behavior of the cells at the time that they were evaluated. Our objective was to compare the results of the samples with the control groups, this variation was considered as part of the statistical analyses and the conclusions were supported. Based on the results, Figure 9 presents a proposed mechanism of the effects of DTP, digested albumin, glutelin and pure peptides in the three experimental approaches analyzed in this paper: (1) prevention of adipogenesis (experiments I and II), (2) prevention of inflammation (experiment III) and (3) inhibition of established inflammation (experiment IV). In summary, the different treatments had effects in every marker related to fibroblast differentiation in adipocytes, thus preventing the adipogenesis process. In addition, DTP and digested glutelin were most effective on reducing markers related with adipogenesis, in both, prevention and inhibition of inflammation. Moreover, DTP, digested albumin and glutelin were most effective on prevention of inflammation, DTP showing the best results.

We are reporting, for the first time, an evaluation of the effects of digested proteins from chia seeds on preventing adipogenesis and also the prevention and inhibition of inflammation on adipocytes and additionally, suggested a mechanism of action. This study showed that DTP, digested albumin, digested glutelin or pure peptides, NSPGPHDVALDQ (Pep1) and RMVLPEYELLYE (Pep2), had a significant effect on preventing adipogenesis by reducing the expression of PPARγ. Consequently, all proteins related to this pathway were less expressed, reducing the expression of markers related to the differentiation of fibroblasts into adipocytes and the accumulation of lipids inside the cells. Furthermore, in mature adipocytes, the use of digested proteins was more effective to reduce adipogenesis and lipogenesis during prevention. DTP and digested albumin and glutelin showed the best results against markers related to prevention of inflammation. The inhibition of PPARγ blocked NF-κB expression, then inflammation was reduced. When the digested proteins were used to inhibit inflammation already establish, DTP stood out with the best results.

## 5. Conclusions

In conclusion, these findings suggest the ability of proteins and peptides from chia seed to block PPARγ, causing a regulation of both, adipogenesis and inflammation. In general, total protein (as a combination of proteins), as well as glutelin, showed better results against adipogenesis and inflammation in adipocytes, indicating that glutelin may be one of the main proteins responsible for the effects observed with the total chia seed protein. Confirming these findings, we observed that pure peptides derived from glutelin also demonstrated beneficial anti-adipogenic results in in vitro and additionally in silico analysis. These results are promising and allow understanding the mechanisms related to the beneficial effects of chia seed consumption against obesity demonstrated in other studies with rats and humans. In addition, there is a need for clinical studies with digested proteins from chia seed to confirm the results found in this investigation. These results also suggest the value of digested chia protein fractions, including pure peptides, against adipogenesis and its associated complications by regulation of PPARγ.

## Data Availability

Data sharing not applicable.

## Supplementary Materials

The following are available online at https://www.mdpi.com/2072-6643/13/1/176/s1, Figure S1: Samples preparation; Figure S2: Effect of digested total protein (1 mg/mL), digested albumin and glutelin (1 mg/mL) and the pure peptides Pep1 (NSPGPHDVALDQ) and Pep2 (RMVLPEYELLYE) (100 μM) on 3T3-L1 cell viability. Analyzed by CellTiter 96 one proliferation assay kit- MTS (Promega Corporation, Madison, WI, USA); Figure S3: Effect of digested total protein (1 mg/mL), digested albumin and glutelin (1 mg/mL) and the pure peptides Pep1 (NSPGPHDVALDQ) and Pep2 (RMVLPEYELLYE) (100 μM) added during the differentiation of adipocytes on lipids accumulation; Table S1: The effects of either digested total protein, or digested albumin, or glutelin, or pure peptides on cytokines secretion; Table S2: Estimated free energy binding (EFE) and chemical interactions among the peptides present in digested total protein and digested albumin and glutelin from chia seeds.
