# Supplementary material for: Protein Digests and Pure Peptides from Chia Seed Prevented Adipogenesis and Inflammation by Inhibiting PPARγ and NF-κB Pathways in 3T3L-1 Adipocytes
Source: Nutrients. 2021 Jan 8;13(1):176. doi: 10.3390/nu13010176 (PMC7826547; doi:10.3390/nu13010176)
Supplement: Supplementary file 1 [file nutrients-13-00176-s001.pdf]

Protein digests and pure peptides from chia seed prevented adipogenesis and its inflammation by inhibition of PPARγ and NF-κB pathways

Mariana Grancieri, Hercia Stampini Duarte Martino and Elvira Gonzalez de Mejia

Supplementary material

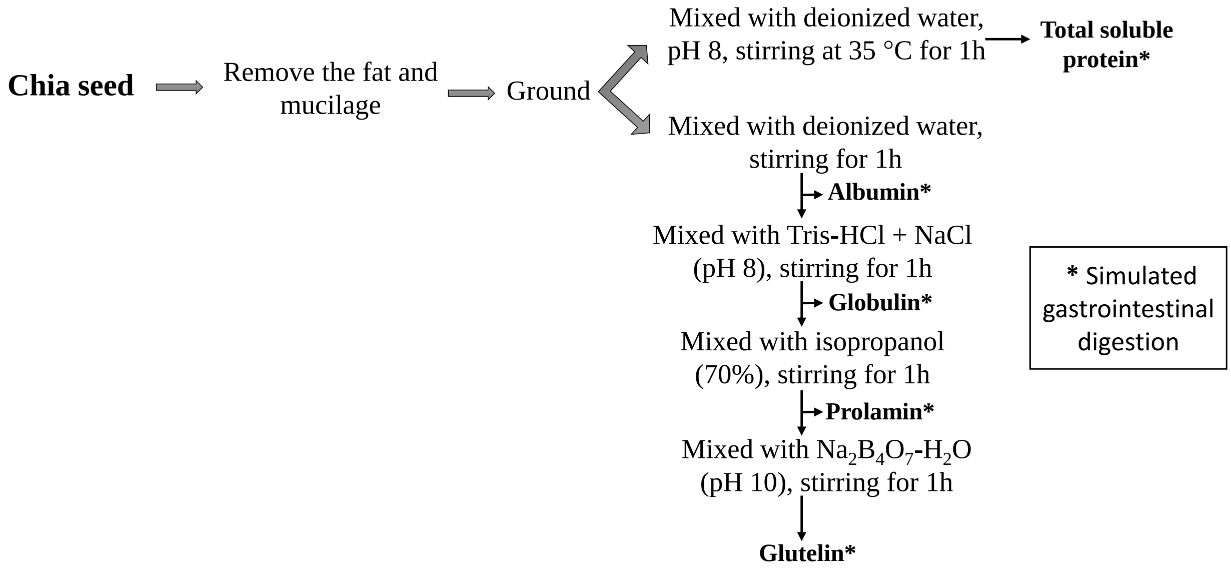

Figure S1. Samples preparation

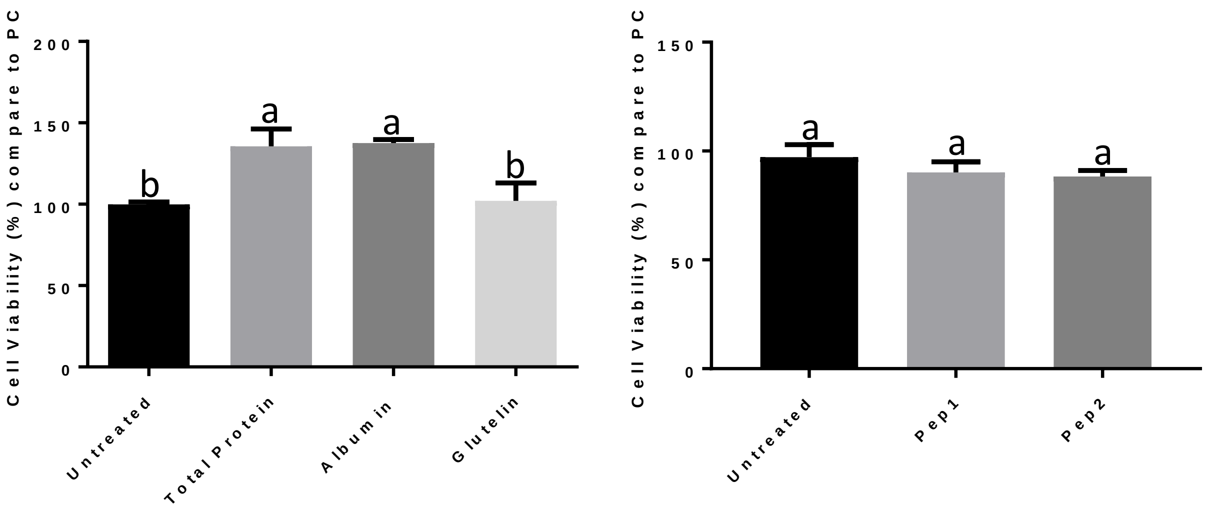

Figure S2. Effect of digested total protein (1 mg/ml), digested albumin and glutelin (1mg/ml) and the pure peptides Pep1 (NSPGPHDVALDQ) and Pep2 (RMVLPEYELLYE) (100µM) on 3T3-L1 cell viability. Analyzed by CellTiter 96 one proliferation assay kit- MTS (Promega Corporation, Madison, WI, USA).

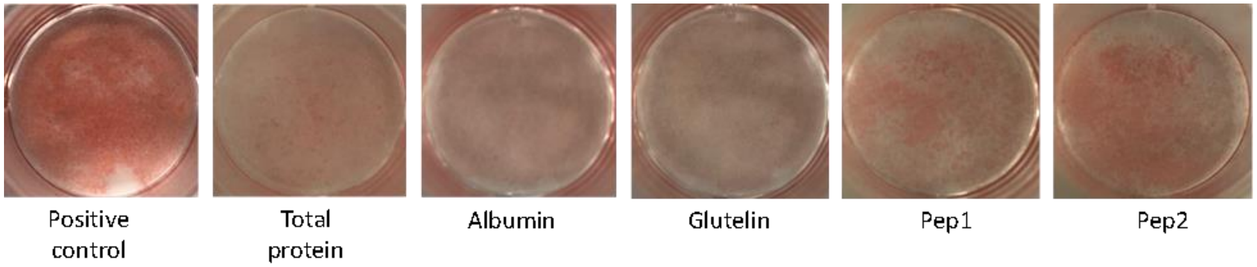

Figure S3. Effect of digested total protein (1 mg/ml), digested albumin and glutelin (1mg/ml) and the pure peptides Pep1 (NSPGPHDVALDQ) and Pep2 (RMVLPEYELLYE) (100µM) added during the differentiation of adipocytes on lipids accumulation.

Table S1. The effects of either digested total protein, or digested albumin, or glutelin, or pure peptides on cytokines secretion.

Experiments	Cytokines	Treatment groups							
		Untreated	CM	PC	Total protein	Albumin	Glutelin	Pep1	Pep2
Adipocytes during differentiation receiving DTP and digested albumin and glutelin (**Experiment 1**)	IL-6			150±15.6^{a}	149.9±5.2^{a}	121.1±4.9^{ab}	107.3±4.8^{b}		
	IL-10			108.1±11.1^{a}	112.9±15.1^{a}	110.9±15.3^{a}	104.9±5.2^{a}		
	IL-12			72.9±4.9^{a}	81.8±2.5^{a}	72.9±4.9^{a}	72.9.9±0.1^{a}		
Adipocytes during differentiation receiving pure peptides (**Experiment 2**)	IL-6			10.8±0.7^{a}				8.7±0.1^{b}	7.1±0.7^{b}
	IL-10			35.1±2.4^{a}				31.6±1.3^{b}	29.5±1.5^{b}
	IL-12			13.5±0.8^{a}				13.5±0.1^{a}	12.6±0.8^{a}
Inflammation prevention in mature adipocytes stimulated with CM (**Experiment 3**)	IL-6	154.6±52.3^{c}	24197±677^{a}	8721±1161.7^{b}	8462±1392^{b}	7330.6±2774.8^{b}	7871.3±1551^{b}		
	IL-10	30.1±0.5^{b}	156.4±8.3^{a}	24.4±0.5^{b}	26.2±1^{b}	27.2±0.5^{b}	28.7±2.5^{b}		
	IL-12	85.4±2.6^{a}	71.2±2.5^{a}	76.5±5^{a}	87.3±5.2^{a}	81.6±7.6^{a}	91±0.1^{a}		
Inflammation inhibition in mature adipocytes stimulated with CM (**Experiment 4**)	IL-6	139±10.2^{a}	23148.4±167.5^{a}	10608.2±405.4^{a}	8475±1048^{a}	8479.8±1339.7^{a}	8579.3±1117.5^{a}		
	IL-10	30.1±0.5^{b}	156.4±8.4^{a}	33.1±1.6^{b}	36.5±3.3^{b}	29.4±0.5^{b}	32.3±0.5^{b}		
	IL-12	85.4±2.6^{b}	71.2±2.5^{b}	121.9±11.3^{a}	138.1±0.1^{a}	119.8±8.4^{a}	127.8±2.9^{a}		

Experiment 1: Effects of digested total protein (DTP) and digested protein fraction (DPF) in 3T3-L1 adipocytes during the differentiation process. **Experiment 2**: Effects of pure peptides NSPGPHDVALDQ (Pep1) and RMVLPEYELLYE (Pep2) in 3T3-L1 adipocytes during the diferentiation process. **Experiment 3**: Effects of DTP and DPF for prevent of inflammation in mature adipocytes stimulated with conditionated media from inflamed macrophages. **Experiment 4**: Effects of DTP and DPF for inhibition of inflammation in mature adipocytes stimulated with conditionated media from inflamed macrophages. Different letter per row means statistically different between the proteins (by ANOVA and post-hoc Tukey-test). CM: conditioned media; PC: Positive control

Table S2. Estimated free energy binding (EFE) and chemical interactions among the peptides present in digested total protein and digested albumin and glutelin from chia seeds.

Peptides	PPARγ		MAGL		FAS	
	EFE	Interacting amino acid residues	EFE	Interacting amino acid residues	EFE	Interacting amino acid residues
Pharmacological control*	-8.5	GLU A: 295; PHE A: 226; ALA A: 292; ILE A: 325; ILE A: 296; ILE A: 326; MET A: 329; GLY A: 344; LEU A: 228; GLU A: 343; SER A: 342; LEU A: 340; ILE A: 341; LEU A: 333; LEU A: 330; ARG A: 288	-7.4	HIS B: 2475; SER B: 2473; MET B: 2444; SER B: 2500; SER B: 2499; PRO B: 2504	-5.5	ALA A: 2276; HIS B: 2283; GLN B: 2432; THR B: 2434; SER B: 2281; LEU A: 2279; TYR A: 2425; PHE A: 2418; ARG A: 2421; ARG A: 2275
Total Protein						
HYGGPPGGCR	-7.0	ARG A: 280; ARG A: 288; GLU A: 343; GLU A: 295; GLU A: 298; SER A: 225	-7.5	ASN A: 215; GLN A: 212; ALA A: 163; ILE A: 211; ALA A: 151; LYS A: 160; VAL A: 217; ALA A: 156; MET A: 123; SER A: 122; HIS A: 269	-8.3	LEU A: 2231; THR A: 2230; TYR A: 2288; LYS B: 2436; LEU A: 2279; SER B: 2281; SER B: 2284; ASP B: 2280; ARG A: 2275
SPPHGAGLGGGMT	-6.3	ASN A: 375; ARG A: 234; THR A: 238; HIS A: 425; LEU A: 377; SER A: 428; LYS A: 434; LYS A: 373; ALA A: 376	-6.7	LYS A: 160; PRO A: 153; ALA A: 151; ALA A: 156; SER A: 176; SER A: 155; LEU A: 184; SER A: 241; ILE A: 179; ALA A: 51	-6.8	PHE A: 2418; ARG A: 2421; SER A: 2422; ALA A: 2277; ARG A: 2275; LYS B: 2436; SER B: 2284; TYR A: 2288; CYS A: 2292; GLN B: 2432; GLU A: 2227; TYR A: 2425; ALA B: 2287
LPVFGLAAEGNVVTYLH	-7.7	GLU A: 427; ASP A: 380; ALA A: 231; TYR A: 219; ILE A: 223; PRO A: 227; SER A: 225	-6.0	LYS A: 206; PRO A: 172; VAL A: 170; CYS A: 208; LEU A: 167; ALA A: 164; LYS A: 160; SER A: 218; ARG A: 222; ARG A: 219; ASN A: 215; GLN A: 212; SER A: 91	-5.9	LYS B: 2436; HIS B: 2283; THR B: 2434; ARG A: 2421; THR A: 2230; PRO A: 2229; ARG A: 2275; GLU A: 2227; TYR A: 2288; CYS A: 2292; ALA A: 2276

TPPGVGGFPWGGAMLGAQ	-7.0	ASN A: 424; GLU A: 427; SER A: 382; GLN A: 420; LYS A: 216; ARG A: 212; ALA A: 231; ALA A: 235; TYR A: 219	-5.8	SER A: 91; ASP A: 92; ASP A: 26; LEU A: 167; ASN A: 168; ALA A: 164; ALA A: 163; ILE A: 211; LEU A: 214; LYS A: 160; SER A: 218; GLN A: 212	-5.3	PRO A: 2229; LEU A: 2231; CYS A: 2292; GLN A: 2295; ASP A: 2291; ALA A: 2276; ARG A: 2275; PRO A: 2278; ASP B: 2280; SER B: 2281; LYS B: 2436; ARG A: 2421; LEU A: 2279; TYR A: 2425; HIS B: 2283; GLU B: 2316; SER A: 2284; HIS A: 2283
APSPPVLGPP	-7.1	GLU A: 295; SER A: 225; PHE A: 287; HIS A: 466; GLN A: 294; THR A: 297	-6.2	ALA A: 164; LYS A: 160; LEU A: 214; ASN A: 215; ILE A: 211; GLN A: 212; SER A: 91	-9.8	TYR A: 2269; THR A: 2230; LEU A: 2231; TYR A: 2288; ARG A: 2275; GLN B: 2432; HIS B: 2283; ARG A: 2421; PHE A: 2418; SER A: 2281; SER B: 2284; TYR A: 2425
NPGGGAPSGH	-5.9	ARG A: 288; GLU A: 295; ALA A: 292; ILE A: 326; MET A: 329; LEU A: 228	-6.8	ALA A: 51; MET A: 123; SER A: 122; CYS A: 242; LEU A: 241; LEU A: 205; ILE A: 179; SER A: 176; PHE A: 159	-5.2	ALA A: 2361; GLU A: 2364; TYR A: 2424
LHNGGPGGEY	-7.1	LEU A: 228; MET A: 329; ALA A: 292; GLU A: 295; ARG A: 288; ILE A: 326; MET A: 348; ILE A: 341; CYS A: 285; SER A: 342; GLN A: 294; HIS A: 466; PHE A: 287; VAL A: 290	-7.2	HIS A: 121; HIS A: 269; MET A: 123; SER A: 122; SER A: 155; ALA A: 151; SER A: 218; LYS A: 160	-6.7	PRO A: 2229; CYS A: 2292; ARG A: 2275; TYR B: 2433; GLN B: 2432; SER B: 2281; ARG A: 2421; HIS B: 2283; ASP A: 2280; GLN B: 2320; TYR A: 2425; LEU A: 2279
DVNPGGPAPHPPWLSVNDL	-7.4	ALA A: 213; HIS A: 217; GLY A: 305; PRO A: 304; SER A: 302; GLU A: 295; ILE A: 223; PRO A: 227; LYS A: 232; LYS A: 224; TYR A: 219; LYS A: 216; ALA A: 215	-6.2	VAL A: 166; VAL A: 207; LEU A: 167; ASN A: 168; CYS A: 208; LYS A: 160; LEU A: 150; ALA A: 156; VAL A: 217; ILE A: 211; ALA A: 164; GLN A: 212; SER A: 91	5.1	
TGPPRPALVFPHAVVP	-5.7	ILE A: 223; ASP A: 220; LYS A: 216; ARG A: 212; TYR A: 219; LYS A: 232; ILE A: 236; ALA A: 235	-4.9	LEU A: 214; ASN A: 215; LYS A: 160; ILE A: 211; ASP A: 92; CYS A: 208; LEU A: 167; ARG A: 219	-8.2	SER B: 2284; SER B: 2281; LYS B: 2436; ALA A: 2277; HIS B: 2283; ARG A: 2275; GLN B: 2432; ALA A: 2276; TYR A: 2288; CYS A: 2292; LEU A: 2231; THR A: 2230; GLN A: 2272; PRO A: 2229

NSSAQYSDPFLALH	-6.6	LYS A: 232; ILE A: 223; THR A: 229; TYR A: 219; ALA A: 231; ARG A: 212; PRO A: 426; GLU A: 427; HIS A: 425; GLU A: 378; ARG A: 234; ASN A: 375; LYS A: 230; ASP A: 380	-5.2	LYS A: 160; SER A: 218; LEU A: 214; ARG A: 219; ARG A: 222; ASN A: 215; ASP A: 92; SER A: 91; ILE A: 211; LEU A: 167	-3.1	TYR B: 2288; ASP A: 2280; LEU A: 2279; SER B: 2281; ASP B: 2280; THR B: 2434; SER B: 2284; TYR A: 2425; ALA B: 2287; HIS B: 2283; LYS B: 2436; ARG A: 2275; ALA A: 2276; TYR A: 2288; CYS A: 2292; PRO A: 2229; VAL A: 2296; LEU A: 2231
			Albumin			
THPTGPAGAGPPMSL	-5.7	TYR A: 219; ILE A: 223; LYS A: 232; ALA A: 235; GLU A: 378; LYS A: 230; ARG A: 234	-6.0	VAL A: 207; CYS A: 208; VAL A: 89; SER A: 91; GLN A: 212; ASN A: 215; ASP A: 92; ARG A: 219; ILE A: 211; LYS A: 160; LEU A: 214; SER A: 218; ARG A: 222; GLU A: 221; VAL A: 217; LEU A: 150	-5.6	ASP A: 2291; TYR A: 2288; CYS A: 2292; LEU A: 2231; HIS B: 2283; SER B: 2281; ARG A: 2275; LEU A: 2279; TYR A: 2425
RGSSSPMAGAAGDLPGSVT	-5.8	ASN A: 424; HIS A: 425; GLU A: 378; ALA A: 231; THR A: 241; TYR A: 219; ASP A: 380; LEU A: 379; ARG A: 234; ASN A: 375; ASN A: 335; LYS A: 336; VAL A: 372	-5.3	ARG A: 222; ASN A: 215; SER A: 218; ILE A: 211; ALA A: 163; GLN A: 212; SER A: 91; LEU A: 167; CYS A: 208; LYS A: 206	-2.3	ASN A: 2225; GLN B: 2432; PRO A: 2229; ARG A: 2233
YLGAHPGTAN	-7.5	TYR A: 219; LYS A: 216; GLN A: 420; LEU A: 423; GLU A: 378; GLU A: 427; ASN A: 335; ASN A: 375; ARG A: 234	-7.8	GLN A: 212; ARG A: 219; ASN A: 215; ARG A: 222; SER A: 218; LYS A: 160; ALA A: 151; ALA A: 156; VAL A: 217; LEU A: 205; ILE A: 179; LEU A: 241; MET A: 123; SER A: 122; ALA A: 51; GLY A: 50; TYR A: 194; VAL A: 270; LEU A: 184; SER A: 181	-7.5	ASP A: 2280; LEU A: 2279; HIS B: 2283; ASP B: 2280; SER B: 2281; GLN B: 2432; ARG A: 2275; THR B: 2434; THR A: 2230
SPKDLALPPGALPPVQ	-7.7	THR A: 238; ARG A: 234; ALA A: 231; HIS A: 425; ASP A: 380; TYR A: 219; ILE A: 223; SER A: 225; VAL A: 307; LYS A: 301	-7.3	SER A: 91; GLN A: 212; ASN A: 215; ARG A:219; ILE A: 211; LEU A: 167; LEU A: 171; VAL A: 166; VAL A: 170; CYS A: 208; LYS A: 206; SER A: 175; ASN A: 173	-7.0	ARG A: 2275; HIS B: 2283; GLU A: 2227; ALA A: 2276; TYR A: 2288; THR A: 2230; PRO A: 2229; ASN A: 2225; LEU A: 2223; THR A: 2255; ARG A: 2233
TVPLLWFRQDPTGPLNL	-6.9	N/A	-6.8	NA	12.7	

TGPSPTAGPPAPGGGTH	-9.1	ARG A: 234; LYS A: 230; ALA A: 231; ASP A: 380; ASP A: 381; TYR A: 219; HIS A: 425; GLU A: 378	-7.7	NA	9.7	
AVLPDLTSSSLLKLDK	-6.2	ARG A: 234; TYR A: 219; THR A: 241; ALA A: 231; LYS A: 232; ILE A: 223; PRO A: 227; LYS A: 224; SER A: 225	-5.7	ARG A: 219; ASN A: 215; SER A: 218; LYS A: 160; ILE A: 211; ALA A: 163; LEU A: 167; VAL A: 207; CYS A: 208; LEU A: 174	-3.7	PRO A: 2229; LEU A: 2231; TYR A: 2288; GLN B: 2432; THR B: 2434; SER B: 2281; TYR A: 2425; LEU A: 2279; ASP A: 2280; ARG A: 2275; HIS B: 2283; LYS B: 2436; PRO A: 2278; ARG A: 2421; GLU B: 2316; SER B: 2284
RMVLPTYFSAQLE	-6.8	TYR A: 219; THR A: 229; ASP A: 220; ARG A: 212; SER A: 382; GLN A: 420; ASN A: 424; PRO A: 426; ALA A: 235; LYS A: 230; ARG A: 234	-7.1	SER A: 218; ARG A: 219; ARG A: 222; GLN A: 212; LEU A: 214; LYS A: 160; ILE A: 211; VAL A: 170; VAL A: 207; LEU A: 167; CYS A: 208; PRO A: 172; LYS A: 206; LEU A: 174; ASN A: 173; SER A: 175	-7.1	ARG A: 2233; MET A: 2232; PRO A: 2229; ASN A: 2225; GLN A: 2272; VAL A: 2296; LEU A: 2231; PRO A: 2278; HIS B: 2283; ARG A: 2275
RHMDLPALVFT	-8.9	GLU A: 298; SER A: 225; PRO A: 227; SER A: 342; ARG A: 288; LEU A: 228; SER A: 289; HIS A: 323	-6.9	LYS A: 160; ALA A: 164; ASN A: 215; GLN A: 212; CYS A: 208; VAL A: 89; LYS A: 206; ASN A: 173; PRO A: 172; LEU A: 167	-7.0	LYS B: 2436; THR B: 2434; HIS B: 2283; GLU A: 2227; GLY A: 2228; ARG A: 2233
GPPDGPCTEGA	-6.8	ARG A: 212; LYS A: 216; TYR A: 219; ALA A: 235; ALA A: 231; ALA A: 376	-5.8	LYS A: 160; ALA A: 163; ILE A: 211; ASN A: 215; GLN A: 212; SER A: 91; CYS A: 208; VAL A: 89; GLU A: 86; LYS A: 206; VAL A: 207; LEU A: 167; VAL A: 170	-8.5	ARG A: 2421; ASP B: 2280; PHE A: 2418; ARG A: 2275; LEU A: 2279; TYR A: 2425; SER B: 2281; SER B: 2284; GLN B: 2432; THR B: 2434; ALA A: 2276; TYR A: 2288; THR A: 2230; LEU A: 2231
SGKVEELAELPDL	-7.0	THR A: 238; LYS A: 240; ALA A: 235; THR A: 241; TYR A: 219; SER A: 382; ASP A: 380; ALA A: 231; LYS A: 230; ARG A: 234; GLU A: 378; ASN A: 375	-6.7	GLU A: 86; CYS A: 208; VAL A: 170; LEU A: 167; ILE A: 211; ALA A: 163; GLN A: 212; ASN A: 215; ARG A: 219; LYS A: 160; ALA A: 156; VAL A: 217; PRO A: 153	-7.2	THR B: 2434; SER B: 2281; LYS B: 2436; ASP B: 2280; TYR A: 2425; ARG A: 2275; CYS A: 2292; THR A: 2230; LEU A: 2231; GLU A: 2227

LPGPPATF	-6.1	TYR A: 219; ARG A: 212; ASP A: 380; ASN A: 424; HIS A: 425; LEU A: 379; ARG A: 234; ALA A: 235; ALA A: 231; LYS A: 240; THR A: 238; LYS A: 230; ASP A: 381; SER A: 382; GLN A: 420; LYS A: 216; GLU A: 378	-5.8	ILE A: 211; LEU A: 214; SER A: 218; ARG A: 222; ARG A: 219; GLN A: 212; ASN A: 215	-9.0	CYS A: 2292; TYR A: 2288; THR A: 2230; ALA A: 2276; LYS B: 2436; HIS B: 2283; ARG A: 2275; LEU A: 2279; PHE A: 2418; ASP B: 2280; SER B: 2281; SER B: 2284
			Glutelin			
KKLELTEYLYEDKK	-6.3	LYS A: 301; PRO A: 227; LYS A: 232; SER A: 225; THR A: 229; LYS A: 224; ILE A: 223; TYR A: 219; ASP A: 220; ASP A: 380; ALA A: 231	-6.0	ALA A: 151; GLU A: 221; SER : 218; ARG A: 222; LEU A: 214; ARG A: 98; SER A: 91; LEU A: 167; VAL A: 166; VAL A: 207; CYS A: 208	16.4	
NSPGPHDVALDQ	-5.6	LYS A: 230; ALA A: 235; LYS A: 232; ALA A: 231; TYR A: 219; GLU A: 378; ARG A: 234	-6.4	ARG A: 98; ASP A: 26; SER A: 91; VAL A: 90; VAL A: 95; VAL A: 78; CYS A: 208; ILE A: 211; SER A: 218; LYS A: 160	-7.3	GLU A: 2227; GLY A: 2228; TYR A: 2288; PRO A: 2229; CYS A: 2292; LYS B: 2436; ASP A: 2291; THR B: 2434; ARG A: 2275; ARG A: 2421; ARG B: 2428; TYR B: 2433; ILE B: 2282; HIS B: 2283; SER B: 2281; LEU A: 2279; ASP A: 2280
KDSGHPNGGLFAESSP	-6.0	PRO A: 227; TYR A: 219; LYS A: 232; THR A: 241; ASP A: 380; LYS A: 230; ALA A: 231; ALA A: 235; LYS A: 240	-6.8	ALA A: 164; VAL A: 166; VAL A: 170; ASN A: 215; GLN A: 212; ASP A: 92; ARG A: 219; ARG A: 222; SER A: 218; GLU A: 221; ALA A: 156; ALA A: 151; VAL A: 217	-5.2	ARG A: 2275; ARG A: 2421; SER B: 2281; GLN B: 2432; GLU A: 2227; THR B: 2434; LYS B: 2436; PRO A: 2229; CYS A: 2292; TYR A: 2288; ALA B: 2437; PRO B: 2435
TGPCNGAAPPAAGSVDCL	-6.6	LYS A: 438; LYS A: 434; SER A; 428; GLU A; 427; ASN A: 424; TYR A: 219; THR A: 229; ALA A: 235; LYS A: 240; GLU A: 378; ASN A: 375; LYS A: 230	-5.2	LYS A: 206; ASN A: 173; LEU A: 174; VAL A: 207; CYS A: 208; VAL A: 166; VAL A: 170; LEU A: 167; ILE A: 211; ALA : 163; ASN A: 215; LYS A: 160; SER A: 218; ARG A: 222	-5.8	SER A: 2253; ASN A: 2225; PRO A: 2229; CYS A: 2292; GLU A: 2227; GLN B: 2432; SER B: 2281; ARG A: 2275; PRO A: 2278; TYR A: 2425
QAGAGGPGAAGH	-7.8	SER A: 332; ARG A: 288; SER A: 342; SER A: 225; GLU A: 298; LYS A: 224; ASP A: 220	-6.7	SER A: 218; ASN A: 215; LYS A: 160; THR A: 157; PRO A: 153; ALA A: 151; MET A: 123	-8.3	THR A: 2230; THR A: 2274; ALA A: 2276; TYR A: 2288; CYS A: 2292; THR B: 2434; SER B: 2281; ARG A: 2275; GLN B: 2432; ARG A: 2421; SER B: 2284; TYR A: 2425; LYS B: 2436

TPPNGLGPSGSPGPHAQ	-6.0	SER A: 225; PRO A: 227; ILE A: 223; LYS A: 232; THR A: 229; TYR A: 219; ALA A: 231; ARG A: 234; LYS A: 230; ASP A: 380; LYS A: 240; THR A: 241; ASP A: 243	-5.3	SER A: 91; VAL A: 89; GLN A: 212; ASN A: 215; ARG A: 219; LEU A: 171; VAL A: 207; LYS A: 206; CYS A: 208; LEU A: 174; ASN A: 173; LYS A: 160; ALA A: 163; LEU A: 214; ILE A: 211; PHE A: 159	-6.0	LEU A: 2231; VAL A: 2296; ARG A: 2233; CYS A: 2292; THR A: 2230; TYR A: 2288; ALA A: 2276; ARG A: 2275; ASP B: 2280; TYR A: 2425; LEU A: 2279
RMVLPEYELLYE	-6.9	GLN A: 420; TYR A: 219; LYS A: 224; ILE A: 223; LYS A: 232; THR A: 241; ALA A: 231; ARG A: 234; GLU A: 378; ASP A: 380; HIS A: 425	-7.3	MET A: 123; SER A: 122; SER A: 155; LEU A: 148; LEU A: 213; ALA A: 151; ALA A: 156; LEU A: 214; LEU A: 150; PRO A: 153; SER A: 218; ARG A: 222; LYS A: 160; ALA A: 164; ALA A: 163; ILE A: 211; LEU A: 167; CYS A: 208; VAL A: 207	-5.9	SER B: 2281; GLU A: 2227; LYS B: 2436; TYR A: 2288; THR A: 2230; PRO A: 2229; GLN B: 2432
RLTGTGMGAASSVLP	-7.7	ILE A: 326; CYS A: 285; SER A: 342; PHE A: 287; GLN A: 294; HIS A: 466; LEU A: 468; LEU A: 318; THR A: 297	-6.2	LYS A: 160; SER A: 218; THR A: 157; VAL A: 217; ALA A: 164; CYS A: 208; GLN A: 212; ASN A: 215; ARG A: 219	-4.9	GLN A: 2272; MET A: 2232; PRO A: 2229; THR B: 2434; GLU A: 2227; TYR A: 2288; CYS A: 2292
RHMDLPLSPSSAGP	-5.8	GLN A: 420; ARG A: 212; LYS A: 216; TYR A: 219; ASN A: 424; HIS A: 425; ARG A: 234; LYS A: 230; ALA A: 231; LYS A: 240; THR A: 241; THR A: 229	-5.2	ALA A: 163; ALA A: 164; SER A: 218; LYS A: 160; LEU A: 214; ILE A: 211; LEU A: 167; CYS A: 208; ASN A: 215; GLN A: 212; ARG A: 219; ARG A: 222	56.9	
TTNPGVDADFPPAREL	-5.9	GLU A: 298; SER A: 225; GLU A: 343; PRO A: 227; LYS A: 232; ILE A: 223; THR A: 229; ASP A: 381; ALA A: 231; LYS A: 230; ARG A: 234; THR A: 238	73.2		-4.7	PRO A: 2229; GLU A: 2227; ALA A: 2276: THR B: 2434; LYS B: 2436; CYS A: 2292; THR A: 2274; THR A: 2230; GLY A: 2228; GLN A: 2295
QPADPNQFVYAPDTH	-5.5	SER A: 429; SRG A: 443; ASP A: 396; ASP A: 475	-5.6	ARG A: 222; SER A: 218; LYS A: 160; ALA A: 163; ILE A: 211; CYS A: 208; GLN A: 212; ASN A: 215; SER A: 91	-6.2	GLU A: 2227; PRO A: 2229; LEU A: 2231; LYS B: 2436; ALA A: 2276; GLN B: 2432; SER B: 2281
FAFFEFFELLFAFFT	-8.5	ARG A: 234; ALA A: 231; ASP A: 380; HIS A: 425; TYR A: 219; ASP A: 220; LYS A: 224; PRO A: 227	-6.5	LYS A: 160; ALA A: 164; ALA A: 163; SER A: 218; ARG A: 222; CYS A: 208; LYS A: 206; ALA A: 203; ARG A: 87	9.2	

TDQLGWFLGAAAFPAGQ	-6.4	GLU A: 298; LYS A: 224; GLN A: 294; GLY A: 284; SER A: 342	-5.5	CYS A: 208; VAL A: 207; VAL A: 170; VAL A: 166; ALA A: 163; PHE A: 159; LEU A: 214; LYS A: 160; ILE A: 211; ASP A: 92; GLN A: 212; ARG A: 219	-4.9	GLU A: 2415; THR A: 2255; ARG A: 2233; GLU A: 2227; ARG A: 2275; HIS B: 2283; ARG A: 2421
RGSSPAPSGSELVL	-4.9	GLN A: 420; ARG A: 212; LEU A: 423; PRO A: 426; ASN A: 424; HIS A: 425; GLU A: 427; LYS A: 240; GLU A: 378; LYS A: 230; ASP A; 380; ALA A: 231; TYR A: 219	-5.9	PRO A: 172; ASN A: 173; ILE A: 174; CYS A: 208; SER A: 91; ASP A: 92; GLN A: 212; ARG A: 219; ARG A: 222; SER A: 218; LEU A: 167; VAL A: 170	-7.2	LEU A: 2231; PRO A: 2229; GLU A: 2227; ALA A: 2276; THR A: 2274; PRO A: 2226; CYS A: 2292; LYS B: 2436; SER B: 2281; LEU A: 2279; SER B: 2284; TYR A: 2425; ARG A: 2275; ARG A: 2421
TDWYDPGAFQ	-6.7	ILE A: 232; TYR A: 219; LYS A: 216; ASP A: 220; ILE A: 223; LYS A: 224; SER A: 225; SER A: 221; TYR A: 299; SER A: 302; GLU A: 298	-7.5	LYS A: 160; SER A: 218; ARG A: 222; ARG A: 219; ASN A: 215; LEU A: 214; ILE A: 211; GLN A: 212; ASN A: 173; VAL A: 207; CYS A: 208	-7.3	CYS A: 2292; LEU A: 2231; THR B: 2434; LYS B: 2436; HIS B: 2283; TYR B: 2433; SER B: 2281; GLN B: 2432; ARG B: 2428
HAPGFLSPAHR	-6.2	TYR A: 219; LYS A: 216; ASP A: 380; ALA A: 231; ASN A: 424	-6.5	SER A: 218; ALA A: 163; ILE A: 211; GLN A: 212; CYS A: 208; LYS A: 206; LEU A: 174	-7.5	TYR A: 2288; CYS A: 2292; LEU A: 2231; ALA A: 2276; GLY A: 2228; GLU A: 2227; ARG A: 2275; GLN B: 2432; ASP B: 2280; SER B: 2281; ARG A: 2421; LEU A: 2279; SER B: 2284; TYR A: 2425
LPGPPATF	-6.1	TYR A: 219; ARG A: 212; ASP A: 380; ASN A: 424; HIS A: 425; LEU A: 379; ARG A: 234; ALA A: 235; ALA A: 231; LYS A: 240; THR A: 238; LYS A: 230; ASP A: 381; SER A: 382; GLN A: 420; LYS A: 216; GLU A: 378	-5.8	ILE A: 211; LEU A: 214; SER A: 218; ARG A: 222; ARG A: 219; GLN A: 212; ASN A: 215	-9.0	CYS A: 2292; TYR A: 2288; THR A: 2230; ALA A: 2276; LYS B: 2436; HIS B: 2283; ARG A: 2275; LEU A: 2279; PHE A: 2418; ASP B: 2280; SER B: 2281; SER B: 2284

EFE: Estimated free energy. Docking calculation were carried out using AutoDock Vina. Negative values mean spontaneous reaction.

* Pharmacological control: GW9962 (2-chloro-5-nitro-N-phenylbenzamide), Orlistat, and endocannabinoid 2-arachidonoylglycerol (2-AG), respectively for PPARγ, FAS, and MAGL.
